# Microfibril Associated Protein 5 (MFAP5) Is Related to Survival of Ovarian Cancer Patients but Not Useful as a Prognostic Biomarker

**DOI:** 10.3390/ijms232415994

**Published:** 2022-12-15

**Authors:** Katarzyna Aleksandra Kujawa, Ewa Zembala-Nożynska, Joanna Patrycja Syrkis, Alexander Jorge Cortez, Jolanta Kupryjańczyk, Katarzyna Marta Lisowska

**Affiliations:** 1 Center for Translational Research and Molecular Biology of Cancer, Maria Skłodowska-Curie National Research of Oncology Center, Gliwice Branch, 44-102 Gliwice, Poland; 2 Tumor Pathology Department, Maria Skłodowska-Curie National Research Institute of Oncology, Gliwice Branch, 44-102 Gliwice, Poland; 3 Department of Biostatistics and Bioinformatics, Maria Skłodowska-Curie National Research Institute of Oncology, Gliwice Branch, 44-102 Gliwice, Poland; 4 Department of Pathology and Laboratory Diagnostics, Maria Skłodowska-Curie National Research Institute of Oncology, 02-781 Warsaw, Poland

**Keywords:** prognostic markers, ovarian cancer, MFAP5, microfibril-associated protein 5, MAGP2, microfibril-associated glycoprotein 2, immunohistochemistry, Kaplan-Meier plotter, CSIOVDB, string, cBioPortal

## Abstract

Ovarian cancer (OC) is usually diagnosed late due to its nonspecific symptoms and lack of reliable tools for early diagnostics and screening. OC studies concentrate on the search for new biomarkers and therapeutic targets. This study aimed to validate the *MFAP5* gene, and its encoded protein, as a potential prognostic biomarker. In our previous study, we found that patients with high-grade serous OC who had higher *MFAP5* mRNA levels had shorter survival, as compared with those with lower levels. Here, we used the Kaplan-Meier Plotter and CSIOVDB online tools to analyze possible associations of *MFAP5* expression with survival and other clinico-pathological features. In these analyses, higher *MFAP5* mRNA expression was observed in the more advanced FIGO stages and high-grade tumors, and was significantly associated with shorter overall and progression-free survival. Next, we analyzed the expression of the MFAP5 protein by immunohistochemistry (IHC) in 108 OC samples and tissue arrays. Stronger MFAP5 expression was associated with stronger desmoplastic reaction and serous vs. non-serous histology. We found no significant correlation between IHC results and survival, although there was a trend toward shorter survival in patients with the highest IHC scores. We searched for co-expressed genes/proteins using cBioPortal and analyzed potential MFAP5 interaction networks with the STRING tool. MFAP5 was shown to interact with many extracellular matrix proteins, and was connected to the Notch signaling pathway. Therefore, although not suitable as a prognostic biomarker for evaluation with a simple diagnostic tool like IHC, MFAP5 is worth further studies as a possible therapeutic target.

## 1. Introduction

High-grade serous ovarian cancer (HG-SOC) is the most frequent and most deadly histological type of OC [1,2,3]. It is usually diagnosed at an advanced stage. Standard treatment includes surgical debulking and platinum-taxane chemotherapy [4,5]. Although the majority of patients respond well to the first line treatment; unfortunately, they frequently experience recurrence. Recurrent disease tends to become chemo-resistant and incurable, in spite of new treatment options recently developed (anti-angiogenic therapies, PARP inhibitors) [6,7]. To improve patient outcomes, we need to better understand the biology of this cancer and search for new biomarkers and potential therapeutic targets.

There is some evidence that HG-SOCs are not uniform at the molecular level. Several molecular subtypes were suggested, which are possibly related to patient prognosis [8,9,10]. In our earlier study, using expression microarrays, we identified two subtypes of HG-SOC, showing a distinct gene expression pattern [11]. In particular, they differed by the expression level of 151 Affymetrix probe-sets, corresponding to 96 unique genes. Moreover, we found that these two molecular subtypes of HG-SOC were associated with different survival: patients with higher expression of this set of genes in the tumor had shorter overall survival (OS) than those with lower expression [11]. The majority of genes from this signature were related to extracellular matrix (ECM) structure and function, and cellular adhesion and motility, features known to be involved in the acquisition of invasive and metastatic phenotypes of cancer. Notably, a similar multigene signature was also identified by others in different solid tumors, e.g., pancreatic, breast, and gastric cancer, and was suggested to be associated with greater cancer invasiveness [12]. The discussion on the cell type responsible for expression of these genes (epithelial versus stromal, e.g., cancer-associated fibroblasts, CAFs) is ongoing and far from being resolved. Although intuitively expression of such a “mesenchymal” signature is thought to be linked with CAFs, there is also some experimental evidence that cancer cells themselves can express those genes [11,13].

Our further studies aimed at investigating genes/proteins from the described negative prognostic signature. We performed both validation studies designed to reveal whether some of these genes could serve as prognostic biomarkers, and functional in vitro studies aimed at disclosing their biological role in OC. So far, we have focused on the following genes, being part of our multigene signature: *LOX* [14], *ITGBL1* [15], *FN1*, and *POSTN* [16]. Our recent study was devoted to the *ITGBL1* gene, coding for the poorly characterized Integrin beta-like 1 protein. Our results confirmed that overexpression of ITGBL1 affects OC cells’ migration rate, adhesiveness, and chemoresistance against cisplatin and paclitaxel [15]. In the other study, we evaluated immunohistochemically expression levels of fibronectin (FN1) and periostin (POSTN); the resulting combined FN1&POSTN score proved to be an independent prognostic factor associated with the survival of OC patients [16]. Lysyl oxidase (LOX) expression evaluated by IHC was higher in omental metastases than in primary tumors; however, it was not significantly associated with OS [14].

In the present study, we aimed to validate the prognostic significance of another gene from our multigene signature, coding for the microfibril-associated protein 5 (MFAP5; also known as MAGP2, microfibril-associated glycoprotein 2) [11]. MFAP5’s role in cancer is poorly characterized, although there are some preliminary data concerning its possible role in OC. It is probably engaged in the regulation of angiogenesis and may be associated with survival [13,17]. MFAP5 is also engaged in cytoskeleton structure and function. Thus, it may be speculated that expression level of this protein can affect cell division and proliferation rate, as well as response to anti-cancer treatment, eventually supporting cancer progression. There is currently no specific small-molecule inhibitor of MFAP5, but initial attempts at treating mice with the anti-MFAP5 antibody showed promising results. It caused decreased collagen production by cancer-associated fibroblasts, suppressed intratumoral microvessel leakiness, and enhanced paclitaxel bioavailability in ovarian and pancreatic cancer models [18].

The principal aim of this study was to check the prognostic significance of *MFAP5* in OC using online platforms such as the Kaplan-Meier Plotter and CSIOVDB. We then evaluated MFAP5 immunohistochemically (IHC) in a series of 108 HG-SOC samples and analyzed whether its expression level is associated with the survival of OC patients. We also used several tissue arrays to analyze whether MFAP5 shows differential expression in consecutive stages of OC progression and distinct histological types, as well as in healthy tissues and benign ovarian tumors. Since it has been postulated that HG-SOC may have either ovarian or fallopian epithelial origin, we also analyzed tissue arrays containing normal and inflamed fallopian tube tissue, benign hyperplasia, and different stages of fallopian tube cancer progression. Finally, using cBioPortal, we searched for other genes coordinately expressed with *MFAP5*. The STRING algorithm was used to analyze possible protein interaction networks involving MFAP5.

## 2. Results

### 2.1. Evaluation of Prognostic Value of MFAP5 mRNA Expression Level

**Figure 1:**
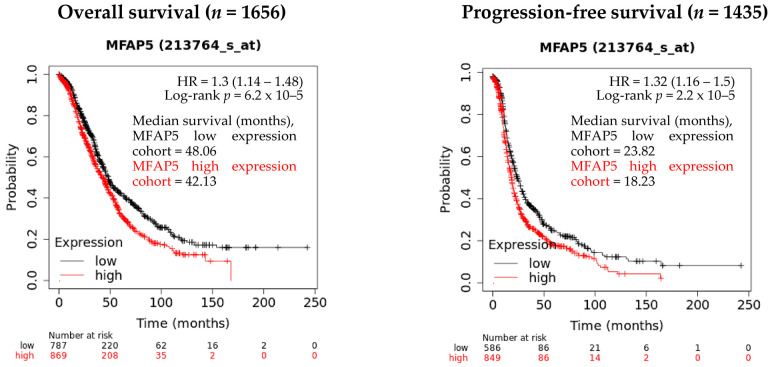
The evaluation of *MFAP5* mRNA expression relevance in regard to OS and PFS using the Kaplan-Meier Plotter database. Low and high expression cohorts stratification was based on the best cutoff value automatically selected by KMP (13 June 2022).

Our previous study, based on gene expression analysis of 100 OC samples showed that the *MFAP5* mRNA expression level was associated with patient survival [11]. Here, we performed survival analysis using two online tools that operate on much larger expression datasets, encompassing several hundreds of OC samples. By using the Kaplan-Meier Plotter (KMP), we confirmed that the *MFAP5* mRNA expression level is associated with the survival of OC patients. Patients with higher *MFAP5* expression had shorter overall survival (OS) (log rank test, *p* = 6.2 × 10−5; FDR = 5%; *n* = 1656) and progression-free survival (PFS) (log rank test, *p* = 2.2e-02; FDR = 2%; *n* = 1435). The *MFAP5* expression was judged ‘low’ or ‘high’ based on the best cutoff value automatically selected by KMP. The median OS was 48.06 vs. 42.13 months in the low *MFAP5* expression cohort and high *MFAP5* expression cohort, respectively. The median PFS was 23.82 months in the low *MFAP5* expression cohort vs. 18.23 months in the high *MFAP5* expression cohort (Figure 1).

**Figure 2:**
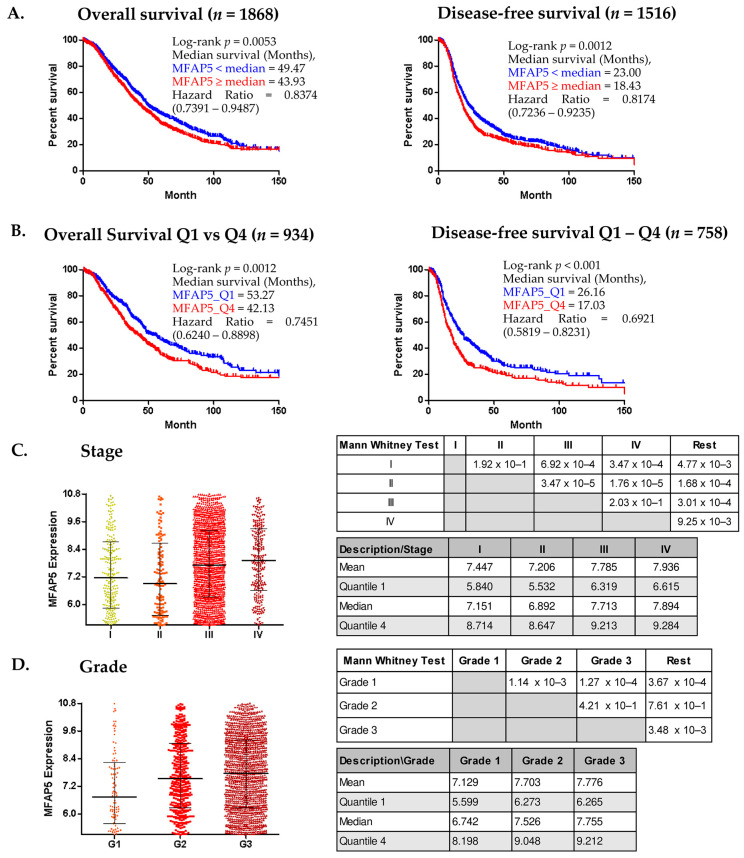
CSIOVDB analyses of *MFAP5* mRNA expression. (**A**,**B**). Kaplan-Meier analysis of survival of ovarian cancer patients stratified according to median *MFAP5* expression (**A**) and to the lower and upper quartile (Q1 & Q4) of *MFAP5* expression (**B**); (**C**,**D**) *MFAP5* expression plot in relation to FIGO stage and tumor grade. Statistical significance evaluated by Mann Whitney U-test; HR calculated for a reference group with lower *MFAP5* expression. (http://csiovdb.mc.ntu.edu.tw/pages/CSIOVDB_mfap5.html; accessed on 13 June 2022).

Similar results were obtained using CSIOVDB, which comprises gene expression data accompanied with OS information for 1868 OC patients and DFS information for 1516 patients. When median *MFAP5* expression was used as a threshold, the median OS was 49.47 vs. 43.93 months in the low expression cohort and in high expression cohort, respectively. The median DFS was 23.00 vs. 18.43 months in the low expression cohort and high expression cohort, respectively (Figure 2A). The differences in survival were more significant when comparing the lower and upper quartile of *MFAP5* expression (Figure 2B).

The CSIOVDB algorithms also enable the analysis of the expression of selected genes according to the molecular subtype of the OC: epithelial-A (Epi-A), epithelial-B (Epi-B), mesenchymal (Mes), stem-like-A (Stem-A), and stem-like-B (Stem-B). According to Tan et al. [19], the inventor of CSIOVDB, Epi-A and Epi-B tumors express epithelial cell markers, such as E-cadherin, EPCAM, various keratin genes, and CD24. Stem-A and Stem B tumors express typical markers for epithelial stem cells: LGR5 and PROM1 (CD133), respectively. The Mes tumor subtype predominantly expresses fibroblastic/mesenchymal genes, such as PDGFRA, **VCAM1**, ZEB1, **TWIST1**; and extracellular matrix genes, including **collagen** and **FN1** (bolded genes are overlapping with our negative prognostic signature). According to Tan et al., Epi-A, Epi-B, and Stem-B subtypes have a better prognosis, while Mes and Stem-A tumors are related to a poorer outcome. Using CSIOVDB, we found that MFAP5 expression was associated with DFS only in patients with the mesenchymal subtype: patients with MFAP5 expression below the median had significantly longer DFS than patients with MFAP5 expression ≥ median; median DFS was 19.35 months vs. 13.38 months, respectively (log-rank test, *p* = 0.0078). There was no significant difference in regard to OS in patients with the mesenchymal subtype of OC (Supplementary Figure S1). In other molecular subtypes, MFAP5 expression was neither associated with OS nor DFS.

Except for the survival analysis, CSIOVDB analyzes the gene expression level in relation to other clinico-pathological features. We found that *MFAP5* mRNA expression was related to the FIGO stage and histological grade of the tumor. Patients with FIGO stage I or II cancer had significantly lower *MFAP5* expression in the tumor than patients with advanced stage (FIGO III or IV) cancer (Figure 2C) (pairwise testing). Similar dependence concerned *MFAP5* expression and tumor grade: patients with well-differentiated (G1) tumors had significantly lower *MFAP5* expression than patients with moderately or poorly differentiated (G2 & G3) tumors (Figure 2D) (pairwise testing). However, there was no correlation between *MFAP5* expression and epithelial-to-mesenchymal transition (EMT) status (Spearman’s rho = 0.17).

In the multivariate Cox regression analysis provided by CSIOVDB, *MFAP5* expression did not prove to be an independent prognostic factor, neither with respect to OS, nor DFS (Table 1 and Table 2). On the contrary, the FIGO stage, debulking status, and serous histology were significantly associated with OS, as well as DFS. Also, the age of patients was significantly associated with DFS. According to the Cox coefficient, we observed that older patients (≥55 years) had a higher risk of recurrence, while advanced stage, serous histology, and suboptimal surgical debulking were factors related to the higher risk of recurrence and death. In particular, patients with advanced stage OC (FIGO III & IV) had a 4 times higher risk of death (HR = 4.06), and serous histology was associated with an over 3 times higher risk of death (HR = 3.67). These two features also significantly increased the risk of recurrence (HR = 3.53 for stage and HR = 2.98 for histology). Notably, a higher risk of recurrence was close to statistical significance for patients with higher *MFAP5* expression in the tumor (*p* = 0.062) (Table 1 and Table 2).

### 2.2. Evaluation of MFAP5 Protein in Ovarian Cancer Samples

**Figure 3:**
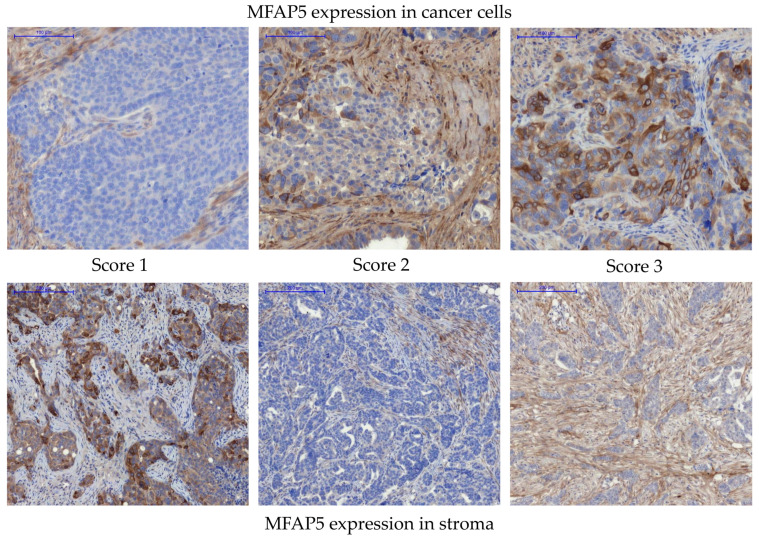
Immunohistochemical detection of MFAP5 in OC samples. Upper panel—representative examples of MFAP5 staining in cancer cells. Lower panel—representative examples of MFAP5 staining in the tumor stroma. The images show different levels of staining, from score 1 (weak expression), through score 2 (moderate expression), to score 3 (strong expression). Pannoramic 250 Flash II Scanner, scale bar: 100 µm.

Since the *MFAP5* mRNA level was found to be associated with OC patients’ survival, we decided to analyze if there is a similar correlation at the protein expression level. MFAP5 expression was evaluated immunohistochemically in the series of 108 OC samples for which we had complete clinico-pathological data, including survival. Based on the recommendation of an experienced pathologist (E.Z.-N.), who assessed preliminary IHC images, we decided to evaluate the MFAP5 expression within two compartments separately: first in cancer cells, then in the tumor stroma (Figure 3 and Supplementary Figure S2). When MFAP5 was assessed in cancer cells, eight samples (7.41%) showed strong expression (score 3), 33 samples (30.55%)—moderate expression (score 2), and 67 samples (62.04%)—weak expression (score 1). MFAP5 expression in the tumor stroma was as follows: seven samples (6.48%) had strong expression (score 3), 20 samples (18.52%)—moderate expression (score 2), and 81 samples (75%)—weak expression (score 1). Subsequently, we merged samples with a stronger MFAP5 expression (score 2 & 3) and compared them to the group with weak MFAP5 expression (score 1) in regard to clinico-pathological features. We found that more than 60% of patients with complete or partial response to chemotherapy had weak MFAP5 expression in cancer cells, whereas patients with tumor progression or no change in tumor size had stronger MFAP5 expression. This correlation was close to statistical significance (exact Fisher test, *p* = 0.079, Supplementary Table S3). However, this result should be considered with caution due to the disproportionate number of samples: there were only three patients with no change/progression and 105 patients with complete or partial response. Notably, these are typical proportions, as the response to the standard first-line chemotherapy is usually very good and the percentage of primary chemoresistant cases is very low.

Additionally, we observed that along with stronger desmoplastic reaction in the tumor, there was a significantly greater percentage of samples with stronger MFAP5 expression, either in cancer cells or in the tumor stroma (exact Fisher test, *p* = 0.035 and *p* < 0.001, respectively) (Supplementary Table S3).

We also observed a greater proportion of stronger MFAP5 staining in grade 4 than in grade 3 tumors (37 vs. 20%, exact Fisher test, *p* = 0.082). Weak MFAP5 staining was more frequent in tumors with stronger inflammatory infiltration, in comparison with tumors with weak inflammatory infiltration (89 vs. 70%, exact Fisher test, *p* = 0.072). These associations were close to statistical significance.

There was no significant difference in MFAP5 expression in relation to other clinico-pathological factors (Supplementary Table S3).

### 2.3. Prognostic Significance of MFAP5 Protein in Advanced Ovarian Cancer

**Figure 4:**
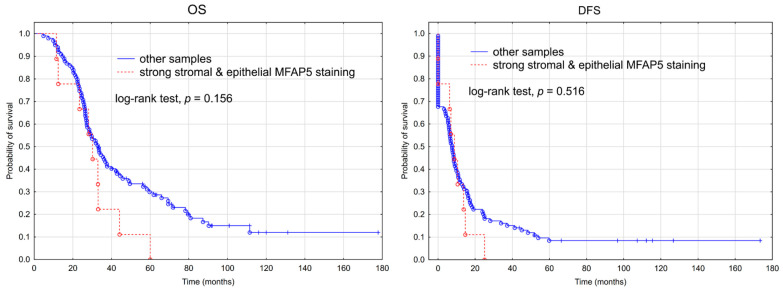
Kaplan-Meier analysis of overall survival (OS) and disease-free survival (DFS) in 108 patients with advanced ovarian cancer stratified by stronger MFAP5 expression (IHC score 2 or 3) both in the stromal and epithelial compartment vs. the rest of samples.

We performed survival analysis in regard to the immunohistochemically assessed MFAP5 expression level. When MFAP5 expression was considered separately either in cancer cells or in the tumor stroma, we observed no significant association neither with OS nor DFS (Supplementary Figure S3). When we selected all samples having stronger MFAP5 expression (score 2 and 3) both in the stromal and epithelial compartment (*n* = 9), and compared them to the rest of the samples (*n* = 99), a slight trend toward worse OS in the first group was visible; however, it was not statistically significant (Figure 4).

### 2.4. Evaluation of MFAP5 Protein in Ovarian Tissue Array

The series of cases described in the previous section were very homogenous, consisting only of advanced, high-grade serous ovarian cancers. Here, we wanted to analyze MFAP5 expression in a more diverse spectrum of ovarian samples. For this purpose, we used commercially available tissue arrays containing normal, benign, borderline, and malignant samples, representing different FIGO stages and histological types (Supplementary Figures S4–S7, Tables S4–S7).

**Figure 5:**
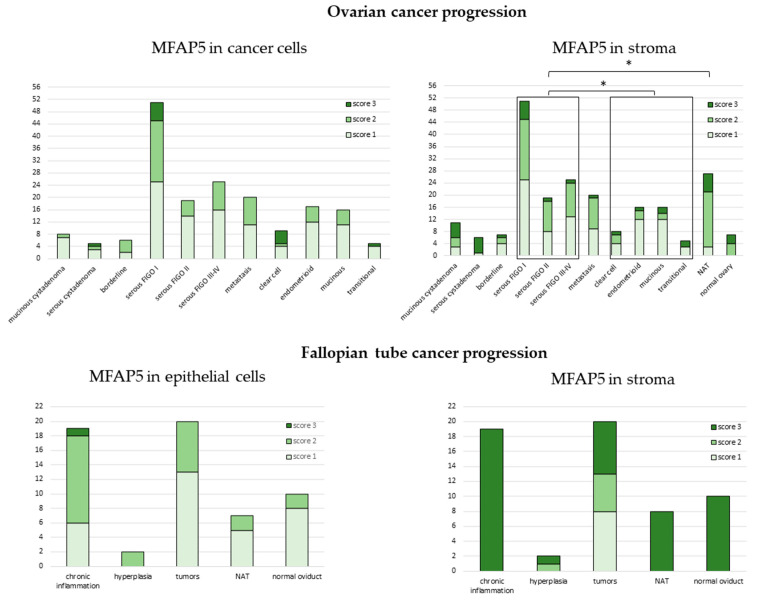
Expression of MFAP5 in tissue arrays. The y axis represents number of samples. NAT—normal tissue adjacent to the tumor. *−denotes *p* < 0.05.

We observed significant differences in MFAP5 staining between serous and non-serous tumors (exact Fisher test, *p* = 0.029; Figure 5, right upper panel). The moderate and strong stromal MFAP5 staining was more frequent in serous than in non-serous tumor samples (OR = 2.36, 95% CI: 1.12 to 4.99, *p* = 0.025).

There was also a significant difference in MFAP5 staining between serous tumors vs. tumor adjacent normal tissue (NAT) (exact Fisher test, *p* = 0.003). Stronger stromal MFAP5 staining was more frequently observed in NAT than in serous tumors (OR = 7.51, 95% CI: 2.12 to 26.63, *p* = 0.002).

There were no significant differences in MFAP5 staining neither between different FIGO stages, nor between distinct histological types when analyzed separately. There was also no difference between normal, benign, borderline, or malignant tissues (Supplementary Figures S8–S10).

Strikingly, on tissue arrays, stronger MFAP5 staining was more frequently observed in the stromal than in the epithelial compartment (OR = 1.89, 95% CI: 1.26 to 2.82, *p* = 0.002). This is opposite to the series of 108 HG-SOC samples first analyzed and indicates that these two series of ovarian cancers differ from each other.

### 2.5. MFAP5 Expression in Fallopian Tube Samples

It has been postulated that ovarian cancers have mixed histological origin, some of them originating from the fallopian tube epithelium. Thus, we also analyzed MFAP5 expression in the tissue array with different stages of fallopian tube neoplasia, from normal and inflamed epithelium, through benign hyperplasia, to cancer (Supplementary Figures S7 and S11, Table S7).

In all fallopian tube samples, we observed the tendency for stronger MFAP5 staining more frequently present in the stromal than epithelial compartment; this was similar to ovarian tissue arrays. Surprisingly, strong stromal MFAP5 expression was prevalent in non-cancerous fallopian tube samples, while not in cancer samples. Among the latter, there were eight samples with weak, five with medium, and seven with strong MFAP5 expression (Figure 5, lower right panel).

### 2.6. Functional Network of MFAP5 Protein

In the next step, we used the cBioPortal platform to search for genes that are coordinately co-expressed with *MFAP5*. We found 71 co-expressed genes (Spearman correlation rho > 0.5). Notably, 35 of these were overlapping with our previously identified 96-gene negative prognostic signature [11] (Supplementary Table S1). The majority of cBioPortal co-expressed genes were coding for extracellular matrix (ECM) components, both of structural and functional significance.

**Figure 6:**
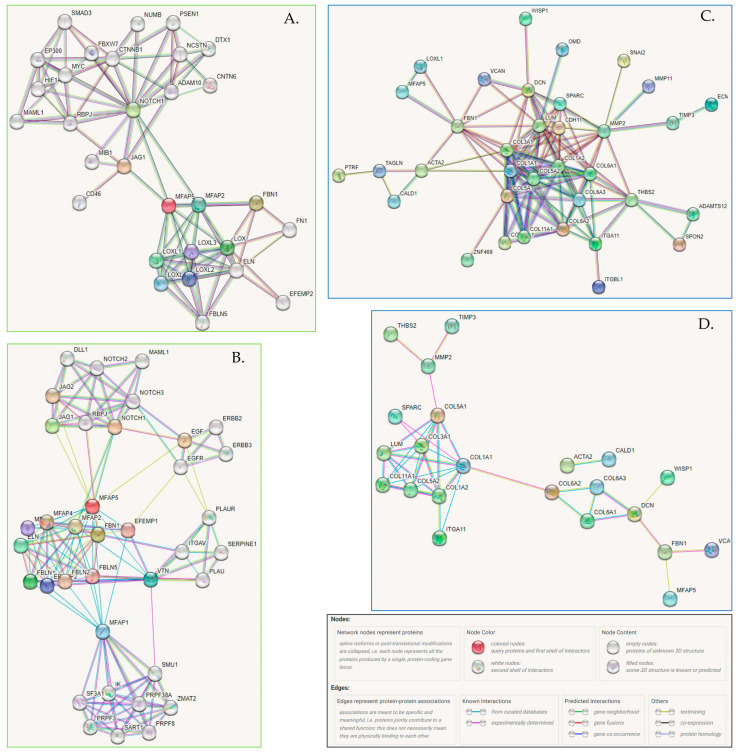
(**A**,**B**) The association network for MFAP5 protein generated by the STRING (v11.5) algorithm. (**A**) STRING settings: full network, all interaction sources (textmining, experiments, databases), first shell—max. 50 interactions, second shell—max. 20 interactions, the cutoff for showing interaction links set to highest confidence (0.900). Edges indicate both functional and physical protein associations; line colors indicate the type of evidence for a given interaction. (https://version-11-5.string-db.org/cgi/network?networkId=bxczNZBaRpdh; accessed on 10 June 2022). (**B**) STRING settings: physical interactions (edges indicate that proteins are part of the same physical complex, but not necessarily directly interacting), line color indicates the type of interaction evidence, interaction sources: textmining, experiments, databases; first shell—max. 50 interactions, second shell—max. 20 interactions, the cutoff for showing interaction links set to medium confidence (0.400). (https://version-11-5.string-db.org/cgi/network?networkId=bevdGiCGDLZV; accessed on 10 June 2022). (**C**,**D**) Functional and physical protein associations of MFAP5 and co-expressed proteins. (**C**) STRING settings: input—71 co-expressed genes (cBioPortal) and MFAP5; full network; all interaction sources (textmining, experiments, databases); first shell—none, second shell—none; the cutoff for showing interaction links set to medium confidence (0.700); disconnected nodes—hidden. Line colors indicate the type of evidence for a given interaction. (https://version-11-5.string-db.org/cgi/network?networkId=bZ1EztNZeLhp; accessed on 10 June 2022). (**D**) STRING settings: input—71 co-expressed genes (cBioPortal) and MFAP5; physical interactions (edges indicate that proteins are part of the same physical complex, but not necessarily directly interacting), line color indicates the type of interaction evidence, interaction sources: textmining, experiments, databases; first shell—none, second shell—none, the cutoff for showing interaction links set to medium confidence (0.400); disconnected nodes—hidden. (https://version-11-5.string-db.org/cgi/network?networkId=bf6tlGslE1NO; accessed on 10 June 2022).

To visualize the network of possible protein-protein interactions involving MFAP5 we used the STRING platform. First, we analyzed interactions between MFAP5 and 71 cBioPortal co-expressed genes/proteins. The STRING algorithm predicted a tight network with strong experimental evidence, involving 35 proteins, mostly collagens, proteoglycans, and metalloproteinases. The main MFAP5 interacting partners seem to be lysyl oxidase homolog 1 (LOXL1) and fibrillin 1 (FBN1), which is a structural component of microfibrils. FBN1 emerged as an MFAP5 partner both in functional and physical interactions (Figure 6C,D and Table S2).

We also performed de novo a search for MFAP5 interacting proteins (Figure 6A,B). The STRING algorithm showed again that MFAP5 interacts mainly with ECM proteins, primarily with LOX family members and FBN1. Notably, based on data from curated databases, MFAP5 was shown to be connected to the Notch signaling pathway. This is a key oncogenic pathway and attempts have been made for its therapeutic targeting.

## 3. Discussion

Ovarian cancer is usually diagnosed at the late stage. This is due to the lack of specific symptoms in early stages of the disease, as well as a lack of reliable screening and early diagnosis methods. For these reasons, the majority of OC studies are aimed at finding new diagnostic markers. The remaining studies concentrate on better understanding the biology of OC to find new prognostic and predictive biomarkers, as well as possible therapeutic targets. The far-reaching aim is to broaden treatment options for patients with advanced disease and improve patient outcomes.

We have previously selected, using microarrays, the set of genes possibly related to the worse prognosis in OC patients [11,20]. Among them was *MFAP5*; showing higher mRNA expression measured by Affymetrix HGU133 2.0 Plus arrays associated with worse OS. We performed qRT-PCR validation of this result and confirmed that the pertinent gene was associated with worse OS, while not DFS. Next, we performed qRT-PCR validation on the independent set of OC samples. In this cohort, *MFAP5* mRNA expression levels were insignificantly associated with OS and DFS. However, that previous study had a weakness related to the small validation group consisting of 33 samples only [11].

However, we are convinced that this gene is worth further studies. *MFAP5*, although insignificant in our external validation, was already indicated by others as a possible prognostic marker in OC [13,17]. Thus, we decided to continue validation on a bigger set of samples. Fortunately, there are now several databases available which gather expression data from numerous transcriptomic experiments. We have chosen to re-evaluate this gene using the KM-Plotter and CSIOVDB online tools.

### 3.1. MFAP5 Validation

We obtained encouraging results for *MFAP5*, indicating that higher mRNA expression of this gene was significantly associated with worse prognosis (shorter OS and DFS). Thus, we decided to evaluate MFAP5 expression immunohistochemically in FFPE OC tissues to check whether the protein expression level correlates with survival. IHC is a simple and convenient method for biomarker evaluation in a hospital setting, as it works on easily obtained FFPE tissue samples and is routinely performed in almost every hospital.

Unfortunately, there was no significant association of MFAP5 expression neither with OS, nor DFS. This was contrary to the results obtained by Mok et al., and Leung et al. [13,17]. Mok et al. [17] analyzed MFAP5 expression in the epithelial compartment of the tumor (42 FFPE OC samples in the learning set and 64 element tissue arrays as an independent validation group) and found that higher expression was significantly associated with worse survival. Leung et al. [13] analyzed stromal expression of MFAP5 on a series of 130 FFPE OC samples and found that higher expression was significantly associated with worse OS. The discrepancy between these studies and our results may be due to the use of different antibodies and detection methods. Mok et al. [17] used antibody from Rockland Inc. (Pottstown, PA, USA), which preferentially stains MFAP5 in the epithelial compartment of the tumor, while Leung et al. [13] used the HPA010553 antibody from Sigma-Aldrich Co. (St. Louis, MI, USA), which detects mostly stromal MFAP5. We used the 15727-1-AP (Proteintech Group, Inc., Wuhan, China) antibody, which stains both stromal and epithelial MFAP5. The immunogen used for production of 15727-1-AP was a peptide covering almost the whole MFAP5 sequence (amino acids 27–173), while that used by Rockland Inc. Ab [17] corresponded to the region near the carboxy-terminal end of MFAP5. HPA010553 antibody was produced using a recombinant protein fragment of MFAP5 corresponding to amino acids 25–173 (microfibril-associated protein 5 precursor recombinant protein epitope signature tag (PrEST)). Notably, in our study, we observed a trend toward worse OS when we merged samples with stronger MFAP5 expression in both the stromal and epithelial compartment of the tumor, and compared them against all other samples. However, this result must be taken with caution, as the number of samples with stronger MFAP5 expression was low. Another possible reason for discrepancies between our results and those of others [13,17] could be the fact that our series of 108 FFPE is derived from patients with FIGO IIIC disease only, which is a rather narrow part of the population.

The use of different antibodies could also be the reason for differences in MFAP5 detection in normal and benign vs. cancer tissues. Mok et al. [17] observed low expression of MFAP5 in normal ovarian epithelial cells and benign cysts, but elevated levels in some malignant tumors. Based on the data from the Human Protein Atlas, we assumed that ovarian tissue may be used as a negative control for anti-MFAP5 antibody validation. Indeed, we observed almost no staining in the ovarian stroma in our control sample (Supplementary Figure S13). However, when analyzing MFAP5 expression on ovarian tissue arrays (US Biomax, Inc., Derwood, MD, USA), we observed elevated levels of MFAP5 in some proportion of tissues of every type (normal, benign, and cancer), both in the stromal and epithelial compartment (Figure 5; Supplementary Figures S9 and S10). We used the same antibody for MFAP5 detection in the control samples and tissue arrays; thus, we suppose that this discrepancy arises from technical differences in samples preparation, e.g., different fixation procedures, or (less likely) genetic differences between two populations of patients. The same factors could be also responsible for differences in the MFAP5 staining between our series of FFPE HG-SOC samples and specimens present on tissue arrays (US Biomax, Inc., Derwood, MD, USA). In the latter, stronger MFAP5 staining was more frequently observed in the stromal than in the epithelial compartment, opposite to FFPE samples, despite using the same antibody and antigen retrieval procedure. 

### 3.2. Immunohistochemistry versus qRT-PCR

A reliable biomarker must demonstrate reasonable sensitivity and specificity. Additionally, its detection should be reproducible and simple. As already mentioned, IHC is the most preferred method for biomarker evaluation. It can be routinely used in the majority of hospitals. Thus, it would be of great value to show that MFAP5 can be assessed by IHC and used to predict patient prognosis. However, our IHC results were disappointing as the protein expression showed weaker, or no correlation with patients’ survival than the mRNA expression of this gene.

It should be stressed that IHC evaluation of a given protein must be well-standardized before it becomes a routine clinical test. In the case of newly proposed biomarker proteins, usually there are no properly validated antibodies offered commercially. Thus, researchers are using different antibodies that give discrepant results, and sometimes it is even uncertainif they really detect the protein of interest. It should be underlined that we made a big effort to evaluate several anti-MFAP5 antibodies available (Figures S21–S25). However, the antibody we finally chose for IHC yielded results different from those obtained by others [13,17].

Another question was if a qRT-PCR test can be proposed for assessment of the *MFAP5* expression level. Technically, qRT-PCR is a more demanding method than IHC. It is difficult to obtain reliable results when using FFPE material. Better results could be achieved when using frozen tissue, but this is logistically problematic in an average hospital without easy access to dry ice and low-temperature freezers. Unfortunately, our results indicate that in the case of *MFAP5* even qRT-PCR measurement would not be informative. The difference in the mRNA expression level between the groups with better and worse prognosis was minute and did not allow achieving separable scores. This indicates that not every gene/protein involved in cancer development and progression can be useful as a clinical biomarker.

### 3.3. Large Sample Size and the p-Value Problem

It is worth raising the issue of *p*-value dependence on sample size. It should be remembered that as sample size grows, statistical tests become sensitive even to very small differences between samples. The *p*-value can be close to zero, but it becomes unreliable [21]. This was probably the case with KMP and CSIOVDB in our analysis. In such a situation, it is recommended to look not only at the *p* value but mainly at the effect size [22,23]. In fact, the HR values we observed in KMP and CSIOVDB analyses were low. As a result, it was difficult, or impossible, to obtain clearly separable scores, which could be ascribed to “good prognosis” or “bad prognosis”. It is probably a common trap in many biomarker studies, which start with enthusiastic preliminary results but end up without practical application of the candidate biomarker.

### 3.4. Possible Role of MFAP5 in Ovarian Cancer

Several data suggest that MFAP5 may be involved in ovarian cancer progression. Mok et al. [17] found that the MFAP5 protein promoted in vitro survival of OC, as well as endothelial cells. Additionally, they showed that MFAP5 affects endothelial cells, increasing their motility and invasiveness, via the α_V_β_3_ integrin receptor. They also observed that expression of MFAP5 in the tumor correlates with microvessel density.

Leung et al. [13] showed that MFAP5 causes increased OC cell motility and invasiveness, but did not affect proliferation rate. Our observations were opposite, i.e., MFAP5 overexpression caused decreased motility and slightly enhanced proliferation [24]. The difference may be cell line-specific: we used the OAW42 cell line, while Leung et al. [13] used A224 and ALST cell lines. The histological origin of the majority of OC cell lines is vague. They were established many years ago but were not carefully described at the time. There was no knowledge then that different histological types of OC can originate from distinct tissues and represent completely different entities [25,26,27,28]. OAW42 is described in the Cellosaurus database as serous cystadenocarcinoma while A224 and ALST are described as serous adenocarcinoma. The OAW42 line is relatively popular and has been re-evaluated according to its originally quoted histological origin in several recent studies [29,30]; reviewed in [31]. Most studies confirm serous histology, but not the high-grade type. However, OAW42 cells have mutations in ARID1A and PIK3CA genes, typical for endometrioid and clear-cell cancers; thus, its origin is still unclear. A224 and ALST lack a wider literature background, and their histological origin has been neither confirmed nor denied.

According to CSIOVDB, higher mRNA expression of *MFAP5* was significantly more frequent in advanced FIGO stages and higher grade tumors. Although in the multivariate analysis, this gene has not proven to be an independent prognostic factor; it seems that it is associated with classical clinical prognostic factors in OC, such as stage, grade, debulking status, and/or histological type. Additionally, we observed that stronger IHC staining of MFAP5 was associated with a stronger desmoplastic reaction and serous (vs. non-serous) histological type, the features that are bad prognostic factors by themselves.

cBioPortal and STRING analyses showed that MFAP5 was co-expressed and interacting with many extracellular matrix proteins. MFAP5 is a 25 kDa glycoprotein, which is a component of microfibrils of the extracellular matrix. It has a matrix-binding domain on the C-terminal end, containing seven cysteine residues and an RGD motif on an N-terminal end that binds the α_V_β_3_ integrin [32,33]; reviewed in [34]. It has been shown that the matrix-binding domain has a strong binding preference for tandem EGF-like motifs, which are present, e.g., in fibrillins, fibulins, Jagged1, Jagged2, Delta1, Notch1, and multiple EGF-like domain protein 6 (MEGF6) (reviewed in [34]). This suggests that MFAP5 may play not only structural, but also a cell modulatory role in ECM.

So far, there are no specific inhibitors against MFAP5 except antibody clone 130A, recognizing a common epitope shared between human and murine MFAP5; it was produced and tested by Yeung et al. [18]. These authors observed that the MFAP5 blockade reduced fibrosis, induced tumor vessel normalization and increased paclitaxel bioavailability in mice models of ovarian and pancreatic cancer. These results are very promising, although a risk of side effects must be evaluated, as MFAP5 is also present in the normal stroma. The mechanism by which MFAP5 blockage exerts its effect is unclear. We suppose that these favorable therapeutic effects could be attributed to modulation of Notch signaling. Possibly, MFAP5 blockage-induced modulation of the Notch pathway can provide more desirable effects and lower toxicity than pan-Notch inhibitors (e.g., γ-secretase inhibitors), so far tested [35]. It remains to be clarified how MFAP5 affects tumor vasculature. It was already shown by Albig et al. that MFAP5 can promote sprouting of endothelial cells by suppression of antiangiogenic Notch signaling (notably, in non-endothelial cells MFAP5 activates the Notch pathway) [36]. Taking into account MFAP5 proangiogenic activity, it is also important to check whether and how its targeting may cause interactions with antiangiogenic therapies already used in OC treatment; however, one can expect anenhanced effect on vasculature normalization. 

## 4. Materials and Methods

### 4.1. Tissue Arrays

We used four types of tissue arrays: T112b, OV1005a, BC11115c and UTE601 (US Biomax, Inc., Derwood, MD, USA) (Supplementary Figures S4–S7, Supplementary Tables S4–S7). For preliminary testing of immunohistochemistry parameters, we used test arrays T112b described as “ovary cancer tissue array, with normal tissue control”, containing 12 cases (2 cores per case, total 24 cores). Clinical data included TNM and FIGO staging, histological type and grading. For IHC evaluation of MFAP5 protein expression, we used OV1005a tissue arrays labeled as “ovary disease spectrum (ovarian cancer progression) tissue array”, containing 27 cases of serous adenocarcinoma, 3 mucinous adenocarcinomas, 10 endometrioid adenocarcinomas, 5 transitional cell carcinomas, 10 metastatic ovarian carcinomas, 25 ovarian adenomas, 17 tumor adjacent normal ovary tissues, and 3 normal ovarian tissues (single core per case, total 100 cases/100 cores). Next, we used the BC11115c tissue array containing different histological types of ovarian cancer including 5 clear cell carcinomas, 62 serous carcinomas, 10 mucinous adenocarcinomas, 3 endometrioid adenocarcinomas, 10 lymph node metastatic carcinomas, and 10 tumor adjacent normal ovary tissue. Additionally, we used UTE601 tissue arrays, described as “fallopian tube disease spectrum (fallopian tube cancer progression)”, containing 10 cases of each: adenocarcinoma and inflammation, 1 hyperplastic sample, 4 tumor adjacent normal tissue samples and 5 normal tissues; two cores per each case.

### 4.2. Clinical Samples Used for Survival Analysis

Formalin-fixed paraffin-embedded (FFPE) tissue samples were collected and sectioned (3 µM) at the Maria Skłodowska-Curie National Research Institute of Oncology in Warsaw (Poland). Tissue samples were derived from 108 patients with advanced OC who did not receive neoadjuvant chemotherapy. All patients were diagnosed with stage IIIC OC (according to the Fédération Internationale de Gynécologie et d’Obstétrique; FIGO). The majority of tumors were serous (*n* = 98) and high-grade (106 grade 3 and grade 4 samples, grading evaluated according to: [37]. Ten samples were classified as undifferentiated and only two samples were grade 2. The mean age of the patients was 53.5 ± 10.22 years (range: 29–75 yrs.). The median follow up was 32.85 months (ranging from 4.8 to 177.8 months). Eighty-eight patients died out of the disease. All tumor samples were previously evaluated according to TP53 accumulation [38,39]. The complete clinico-pathological characteristics of the group is given in Table 3.

### 4.3. Immunohistochemistry

Tissue arrays and FFPE tissue sections were treated similarly. The only exception was the initial baking applied to tissue arrays for at least 30 min at 60 °C (Heraeus incubator, Kendro Laboratory Products LP, Hanau, Germany) to remove excess paraffin. Slides with tissue samples were de-paraffinized in xylene and rehydrated in decreasing concentrations of ethanol. Antigen retrieval was performed by boiling in 0.01 M citrate buffer (pH 6.0) in a microwave (Samsung RE-630D; 220 V~50 Hz, 1.15 kW) set at medium power. The buffer with slides was boiled two times for 5 min with 5 min cooling between boiling cycles. Next, slides were allowed to cool down in buffer, then rinsed three times with PBS. Endogenous peroxidase was blocked with 3% hydrogen peroxide, followed by normal horse-blocking serum (2.5%; included in ImmPRESS Anti-Rabbit Ig Reagent Kit, MP-7401, Vector Laboratories, Inc., Burlingame, CA, USA) for 20 min. Then, sections were incubated with primary antibodies at 4 °C for 12 h. We used rabbit anti-human MFAP5 polyclonal antibody (1:400 dilution, 15727-1-AP, Proteintech Group, Inc., Wuhan, China). Sections were rinsed with PBS thrice and incubated for 30 min with secondary antibody conjugated to HRP (concentration 1×, ready to use solution, ImmPRESS Anti-Rabbit Ig Reagent Kit, MP-7401, Vector Laboratories) at room temperature. Immunostaining was performed with 3–3′ diaminobenzidine tetrahydrochloride (DAB), and tissue samples were counterstained with hematoxylin. The sections were examined by light microscopy.

For optimization of the IHC procedure, we used samples of the normal placenta and normal colon tissue for anti-MFAP5 staining. These proteins were also tested within normal ovary tissue. The evaluation of antibodies was described in Supplementary Materials (Supplementary Figures S12–S14).

### 4.4. Pathological and Immunohistochemical Evaluation of Tissue Samples

Pathological assessment of hematoxylin- and eosin-stained tissue sections was performed to confirm previous diagnosis of histological type and grade. Additionally, other features were evaluated, as follows: type of tumor growth (solid, papillary or mixed), angioinvasion (presence of cancer cells within the blood vessels), mitotic activity of cancer cells, inflammatory infiltration, presence of necrosis, calcifications (presence of psammoma bodies), desmoplastic reaction, and anatomical source of the sample. The latter was evaluated based on the presence in the tissue section of peritoneal structures/omental adipose tissue (samples described as P) or ovarian structures (described as O). Cancer samples without any of these structures were described as T (tumor). Desmoplastic reaction was assessed as score 1—single connective tissue fibers, score 2—intermediate quantity of connective tissue fibers, score 3—a large number of connective tissue fibers. Inflammatory infiltration was assessed as weak—small number of inflammatory cells, strong—a large number of inflammatory cells, and moderate—intermediate quantity of inflammatory cells.

After the preliminary assessment of the staining patterns obtained from IHC, we decided to score staining intensity separately in cancer cells and separately in the tumor stroma. For the assessment of MFAP5 expression, a three-stage quantitative scale was used; score 1—was assigned when less than 30% of cancer cells (or connective fibers) were stained, score 2—when 30–60% of indicated structures were stained, score 3—for more than 60% of stained structures. All samples were reviewed and scored by two independent researchers (K.A.K. & E.Z.-N.), including one experienced pathologist (E.Z.-N.). The slides were scanned as whole slide images using a Pannoramic 250 Flash II Scanner (3DHISTECH Kft., Budapest, Hungary).

### 4.5. Statistical Analysis

Statistical analysis was performed using Statistica version 13.1 (TIBCO Software Inc., Palo Alto, CA, USA). Overall survival was calculated from the date of diagnosis to the date of death or last follow-up. Disease-free survival was calculated for patients with complete response to the first-line chemotherapy, as a time without symptoms. Survival data were plotted with the Kaplan-Meier method and the log-rank test was used to compare survival between groups. Associations between protein expression and clinic-pathological variables were studied by exact Fisher test.

### 4.6. Kaplan-Meier Plotter

The Kaplan Meier plotter is the database and online tool that enables a meta-analysis based discovery and validation of survival-related biomarkers [40]. It collects available gene expression data for 21 cancer types, including ovarian (*n* = 2190). An ovarian cancer database includes the following datasets: The Cancer Genome Atlas dataset (TCGA; *n* = 565), and 14 datasets from Gene Expression Omnibus, GSE14764 (*n* = 80), GSE15622 (*n* = 35), GSE18520 (*n* = 63), GSE19829 (*n* = 28), GSE23554 (*n* = 28), GSE26193 (*n* = 107), GSE26712 (*n* = 195), GSE27651 (*n* = 49), GSE30161 (*n* = 58), GSE3149 (*n* = 116), GSE51373 (*n* = 28), GSE65986 (*n* = 55), GSE9891 (*n* = 285), and GSE63885 (*n* = 101). Of note, GSE63885 is the dataset previously published by us [20]. The database is available at https://kmplot.com/analysis/index.php?p=service&cancer=ovar (accessed on 5 June 2022.

For *MFAP5* expression analysis, we have chosen 213764_s_at probe set, the best probe set (according to JetSet [41]) for unambiguous expression estimation using Affymetrix microarray data.

### 4.7. CSIOVDB: A Microarray Gene Expression Database of Ovarian Cancer Subtype

CSIOVDB is a transcriptomic microarray database of 3431 human samples, including carcinoma of the ovary, fallopian tube, and peritoneum, as well as metastasis to the ovary from other sites [8]. The database also comprises samples of stroma and ovarian surface epithelium from a normal ovary and over 400 early-stage ovarian cancers. Additionally, this database offers classification of the tumors according to major OC histological types (clear cell, endometrioid, mucinous, low-grade serous, serous), and molecular subtype (based on: [19]), and specifies the epithelial-mesenchymal transition status for each OC sample. Specified clinico-pathological parameters include tumor grade, surgical debulking status, clinical response and age. The database contains 1868 and 1516 samples with information pertaining to overall and disease-free survival rates, respectively (among these our dataset GSE63885). CSIOVDB offers the multivariate Cox regression analysis, within which analyzed factors and gene expression values are converted to binary states and analyzed as follows (according to personal communication with Tuan Zea Tan): early (FIGO stage I & II) vs. late stage (FIGO stage III & IV); low (G1) vs. high grade (G2, G3); optimal vs. suboptimal surgical debulking; younger (<55 years) vs. older age (≥55 years); non-serous vs. serous histology; low (<median) vs. high (≥median) gene expression. The reference groups used in the model were as follows: FIGO III & IV, grade G2&G3, suboptimal debulking, serous histology, older age (≥55 years), and high gene expression (≥median). In the case of multivariate analysis, CSIOVDB does not show a hazard ratio (coefficient) value for analyzed covariates. We calculated the exponentiated coefficients (exp(Cox coefficient) = hazard ratio), based on data from CSIOVDB. The CSIOVDB database is available at http://csiovdb.mc.ntu.edu.tw/CSIOVDB.html (accessed on 5 June 2022).

### 4.8. cBioPortal

The cBioPortal for Cancer Genomics is an open-access, open-source resource for interactive exploration of molecular profiles and clinical attributes from large-scale cancer genomics projects [42,43]. For ovarian cancer studies, the cBioPortal offers data from the Cancer Genome Atlas (TCGA) Ovarian Serous Cystadenocarcinoma (source data from GDAC Firehose, 600 samples). The cBioPortal was originally developed and is hosted at Memorial Sloan Kettering Institute. The software is now developed and maintained by a multi-institutional team, including the Dana Farber Cancer Institute, Princess Margaret Cancer Centre in Toronto, Children’s Hospital of Philadelphia, The Hyve in the Netherlands, and Bilkent University in Ankara, Turkey. The database is available at https://www.cbioportal.org/ (accessed on 3 June 2022).

### 4.9. STRING: Functional Protein Association Networks

STRING is a database of known and theoretically predicted protein-protein interactions. STRING imports data about experimentally confirmed protein–protein interactions through literature curation, and computationally predicted interactions from: (i) text mining of scientific texts, (ii) interactions computed from genomic features, and (iii) interactions transferred from model organisms based on orthology. STRING imports protein association knowledge from databases of physical interaction and databases of curated biological pathway knowledge. The interactions include direct (physical) and indirect (functional) associations; they stem from computational prediction, from knowledge transfer between organisms, and from interactions aggregated from other (primary) databases (https://string-db.org/, accessed on 5 June 2022) [44]. We used STRING v11.5.

## 5. Conclusions

*MFAP5* mRNA expression is higher in more advanced FIGO stages, as well as in poorly differentiated tumors, and is significantly associated with shorter overall and progression-free survival of OC patients. However, at the protein level, these associations are less clear. Stronger MFAP5 protein expression is associated with stronger desmoplastic reaction and with serous vs. non-serous histology, while there is no significant association with survival, only a trend toward shorter OS in patients with the highest MFAP5 scores. MFAP5 interaction networks involve key oncogenic signaling pathways, e.g., Notch. Thus, although not suitable as a prognostic biomarker for easy evaluation by IHC, based on our results and the literature data, MFAP5 seems worthy of further studies as a possible therapeutic target.

## Figures and Tables

**Table 1:** Multivariate analysis—OS (We calculated HR from the exponentiated coefficients (exp(Cox coefficient) = hazard ratio), based on data from CSIOVDB).

Factor	Details	Cox Coefficient	*p*-Value	Hazard Ratio
**stage**	I, II vs. III, IV; reference III, IV	1.40	<0.001	4.06
**grade**	G1 vs. G2, G3; reference G2 & G3	0.65	0.091	1.92
**surgical debulking**	optimal vs. suboptimal; reference suboptimal	0.20	0.035	1.22
**histology**	non-serous vs. serous; reference serous	1.30	0.009	3.67
**age**	<55 vs. ≥55; reference ≥ 55	0.16	0.076	1.18
***MFAP5***	<median vs. ≥median; reference ≥ median	<0.001	0.998	1.00

**Table 2:** Multivariate analysis—DFS (We calculated HR from the exponentiated coefficients (exp(Cox coefficient) = hazard ratio), based on data from CSIOVDB).

Factor	Details	Cox Coefficient	*p*-Value	Hazard Ratio
**stage**	I, II vs. III, IV; reference III, IV	1.26	<0.001	3.53
**grade**	G1 vs. G2, G3; reference G2 & G3	0.32	0.355	1.37
**surgical debulking**	optimal vs. suboptimal; reference suboptimal	0.25	0.008	1.28
**histology**	non-serous vs. serous; reference serous	1.09	<0.001	2.98
**age**	<55 vs. ≥55; reference ≥ 55	0.18	0.042	1.20
***MFAP5***	<median vs. ≥median; reference ≥ median	0.16	0.062	1.18

**Table 3:** Characteristics of patients and tumor samples.

Characteristics (Total Number)		Number of Samples in Each Category							
**Residual tumor ^1^**	(108)	R0	17	R1	27	R2	21	R3	43
**CHT response (acc. To RECIST^) ^2^**	(108)	CR	74	PR	31	NC	1	P	2
**Histopathological type**	(108)	serous	97	undifferentiated	10	other	1		
**Tumor grade**	(108)	G2	2	G3	76	G4 *	30		
**Platinum sensitivity ^3^**	(108)	Highly sensitive	22	Moderately sensitive	42	Resistant	44		
**p53 accumulation**	(108)	Yes	68	No	40				
**Age**	(108)	≤54 years	56	>54 years	52				

^1^ Residual tumor size: R0 = 0 cm, R1 < 0.5 cm, R2 between 0.5 cm and 2 cm, R2 ≥ 2 cm; ^2^ Chemotherapy (CHT) response, described as clinical status of the patient after first-line treatment: CR—complete response, PR—partial response, NC—no change, P—progression, RECIST—Response Evaluation Criteria in Solid Tumors; ^3^ Tumors were classified as highly sensitive when disease-free survival (DFS) > 732 days, moderately sensitive when 732 days > DFS > 180 days, and resistant when DFS < 180 days; *—classification criteria given by Barber [37].

## Supplementary Materials

The following are available online at https://www.mdpi.com/article/10.3390/ijms232415994/s1.
