# Supplementary material for: Microfibril Associated Protein 5 (MFAP5) Is Related to Survival of Ovarian Cancer Patients but Not Useful as a Prognostic Biomarker
Source: Int J Mol Sci. 2022 Dec 15;23(24):15994. doi: 10.3390/ijms232415994 (PMC9787877; doi:10.3390/ijms232415994)

**Supplementary Materials**


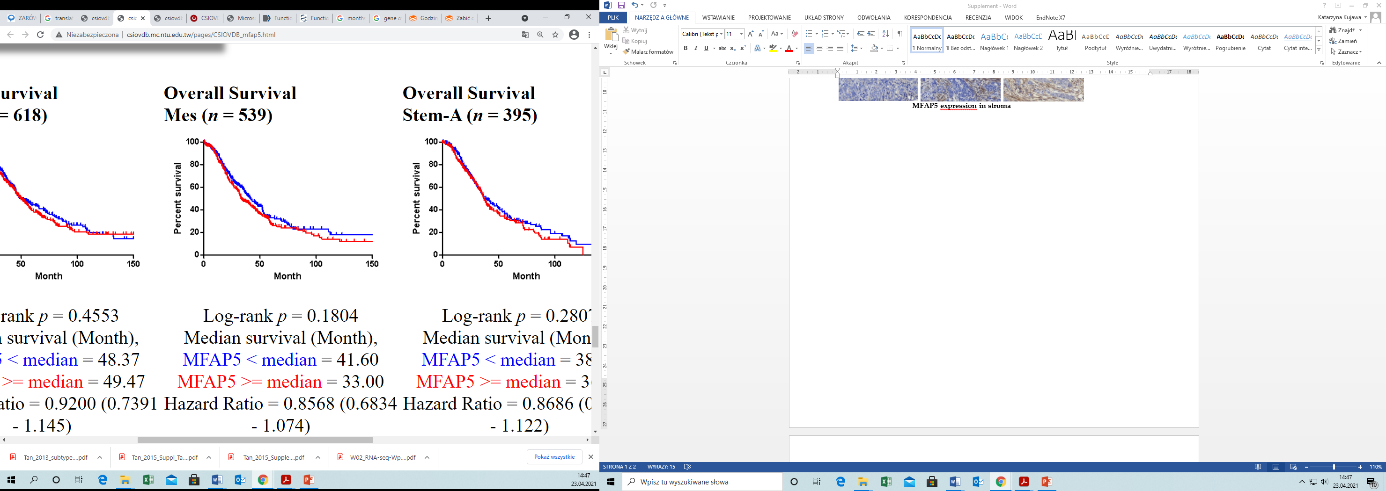

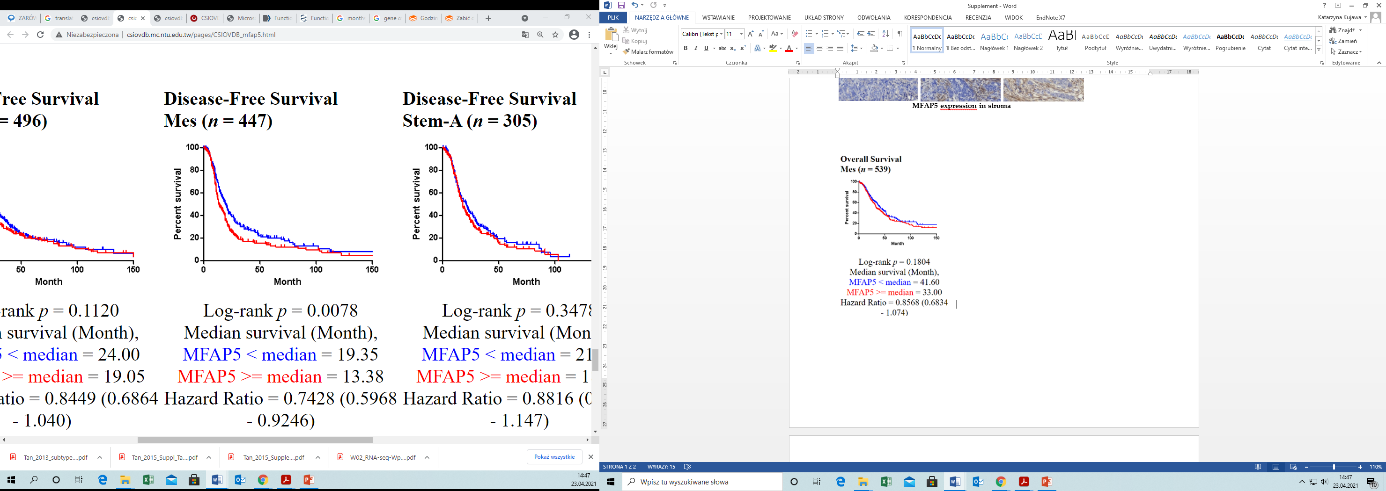


Figure S1. The evaluation of *MFAP5* mRNA expression relevance in regard to OS and DFS in mesenchymal molecular subtype of ovarian cancer patients (http://csiovdb.mc.ntu.edu.tw/pages/CSIOVDB_mfap5.html; 11/06/2021)

**Table S1.** The list of *MFAP5* co-expressed (Spearman’s correalation > 0.5) genes, according to cBioPortal (data from RNA Seq V2 RSEM). Genes which overlap with 96-gene prognostic signature identified previously by us (Lisowska, K. M., M. Olbryt, S. Student, K. A. Kujawa, A. J. Cortez, K. Simek, A. Dansonka-Mieszkowska, I. K. Rzepecka, P. Tudrej, and J. Kupryjanczyk. "Unsupervised Analysis Reveals Two Molecular Subgroups of Serous Ovarian Cancer with Distinct Gene Expression Profiles and Survival." J Cancer Res Clin Oncol 142, no. 6 (2016): 1239-52) are marked blue; (https://www.cbioportal.org/results/coexpression?cancer_study_list=ov_tcga&Z_SCORE_THRESHOLD=2.0&RPPA_SCORE_THRESHOLD=2.0&profileFilter=mrna_U133_all_sample_Zscores&case_set_id=ov_tcga_all&gene_list=MFAP5%253A%2520EXP%253C%253D0%250AMFAP5%253A%2520EXP%253E0&geneset_list=%20&tab_index=tab_visualize&Action=Submit; 24/06/2021).

| No | Correlated Gene | Rank in 96-gene prognostic signature | Cytoband | Spearman's correlation | p-value | q-value |
| --- | --- | --- | --- | --- | --- | --- |
| 1 | *GPR1* |  | 2q33.3 | 0.601 | 1.63e-31 | 2.75e-27 |
| 2 | *OMD* |  | 9q22.31 | 0.599 | 2.73e-31 | 2.75e-27 |
| 3 | ***DCN*** | 1 | 12q21.33 | 0.581 | 4.21e-29 | 2.82e-25 |
| 4 | ***COL10A1*** | 2 | 6q22.1 | 0.577 | 1.25e-28 | 6.31e-25 |
| 5 | *COL1A2* |  | 7q21.3 | 0.576 | 1.79e-28 | 7.19e-25 |
| 6 | ***LUM*** | 3 | 12q21.33 | 0.575 | 2.23e-28 | 7.48e-25 |
| 7 | ***COL1A1*** | 4 | 17q21.33 | 0.574 | 2.93e-28 | 8.42e-25 |
| 8 | ***SPARC*** | 5 | 5q33.1 | 0.573 | 3.73e-28 | 9.38e-25 |
| 9 | ***COL3A1*** | 6 | 2q32.2 | 0.569 | 1.08e-27 | 2.40e-24 |
| 10 | ***COL11A1*** | 7 | 1p21.1 | 0.566 | 2.02e-27 | 4.07e-24 |
| 11 | ***COL5A1*** | 8 | 9q34.3 | 0.565 | 2.79e-27 | 5.10e-24 |
| 12 | ***COL8A1*** | 9 | 3q12.1 | 0.563 | 4.51e-27 | 7.57e-24 |
| 13 | ***MMP2*** | 10 | 16q12.2 | 0.562 | 5.43e-27 | 8.40e-24 |
| 14 | ***GLT8D2*** | 11 | 12q23.3 | 0.558 | 1.54e-26 | 2.07e-23 |
| 15 | ***MMP11*** | 12 | 22q11.23 | 0.558 | 1.54e-26 | 2.07e-23 |
| 16 | *COPZ2* |  | 17q21.32 | 0.557 | 2.00e-26 | 2.51e-23 |
| 17 | ***FBN1*** | 13 | 15q21.1 | 0.556 | 2.95e-26 | 3.50e-23 |
| 18 | *TAGLN* |  | 11q23.3 | 0.554 | 3.95e-26 | 4.42e-23 |
| 19 | *SHISA2* |  | 13q12.13 | 0.552 | 6.77e-26 | 7.18e-23 |
| 20 | *PDPN* |  | 1p36.21 | 0.546 | 2.84e-25 | 2.86e-22 |
| 21 | *ITGA11* |  | 15q23 | 0.545 | 4.03e-25 | 3.86e-22 |
| 22 | ***CALD1*** | 14 | 7q33 | 0.543 | 6.47e-25 | 5.87e-22 |
| 23 | ***CDH11*** | 15 | 16q21 | 0.543 | 6.80e-25 | 5.87e-22 |
| 24 | ***THBS2*** | 16 | 6q27 | 0.542 | 7.00e-25 | 5.87e-22 |
| 25 | ***CTSK*** | 17 | 1q21.3 | 0.541 | 1.07e-24 | 8.64e-22 |
| 26 | ***FNDC1*** | 18 | 6q25.3 | 0.540 | 1.20e-24 | 9.26e-22 |
| 27 | ***ITGBL1*** | 19 | 13q33.1 | 0.537 | 2.65e-24 | 1.98e-21 |
| 28 | ***VCAN*** | 20 | 5q14.2-q14.3 | 0.536 | 2.93e-24 | 2.09e-21 |
| 29 | *ADAMTS12* |  | 5p13.3-p13.2 | 0.536 | 3.05e-24 | 2.09e-21 |
| 30 | *ECM2* |  | 9q22.31 | 0.536 | 3.19e-24 | 2.09e-21 |
| 31 | ***FAP*** | 21 | 2q24.2 | 0.536 | 3.22e-24 | 2.09e-21 |
| 32 | *GALNT5* |  | 2q24.1 | 0.536 | 3.46e-24 | 2.14e-21 |
| 33 | *CREB3L1* |  | 11p11.2 | 0.536 | 3.51e-24 | 2.14e-21 |
| 34 | *C5ORF46* |  | 5q32 | 0.532 | 8.12e-24 | 4.81e-21 |
| 35 | ***AEBP1*** | 22 | 7p13 | 0.529 | 1.48e-23 | 8.53e-21 |
| 36 | ***COL6A3*** | 23 | 2q37.3 | 0.529 | 1.58e-23 | 8.85e-21 |
| 37 | *GREM2* |  | 1q43 | 0.528 | 1.87e-23 | 1.02e-20 |
| 38 | ***TIMP3*** | 24 | 22q12.3 | 0.528 | 2.05e-23 | 1.08e-20 |
| 39 | *LRRC15* |  | 3q29 | 0.527 | 2.52e-23 | 1.30e-20 |
| 40 | ***CTHRC1*** | 25 | 8q22.3 | 0.527 | 2.68e-23 | 1.35e-20 |
| 41 | *AK5* |  | 1p31.1 | 0.526 | 3.22e-23 | 1.58e-20 |
| 42 | *NTM* |  | 11q25 | 0.524 | 5.21e-23 | 2.50e-20 |
| 43 | *SRPX2* |  | Xq22.1 | 0.523 | 5.56e-23 | 2.60e-20 |
| 44 | *FAM155A* |  | 13q33.3 | 0.523 | 5.73e-23 | 2.62e-20 |
| 45 | ***COL12A1*** | 26 | 6q13-q14.1 | 0.523 | 6.15e-23 | 2.75e-20 |
| 46 | *PLPPR4* |  | 1p21.3-p21.2 | 0.521 | 9.18e-23 | 4.02e-20 |
| 47 | ***COL5A2*** | 27 | 2q32.2 | 0.520 | 1.07e-22 | 4.57e-20 |
| 48 | ***INHBA*** | 28 | 7p14.1 | 0.515 | 3.06e-22 | 1.28e-19 |
| 49 | ***VGLL3*** | 29 | 3p12.1 | 0.515 | 3.35e-22 | 1.37e-19 |
| 50 | ***SNAI2*** | 30 | 8q11.21 | 0.515 | 3.53e-22 | 1.42e-19 |
| 51 | ***EDIL3*** | 31 | 5q14.3 | 0.514 | 4.44e-22 | 1.75e-19 |
| 52 | *ACTA2* |  | 10q23.31 | 0.512 | 6.54e-22 | 2.53e-19 |
| 53 | *CCN4* |  | 8q24.22 | 0.512 | 7.20e-22 | 2.73e-19 |
| 54 | *FGF14* |  | 13q33.1 | 0.510 | 1.00e-21 | 3.74e-19 |
| 55 | *LINC00922* |  | 16q21 | 0.510 | 1.05e-21 | 3.83e-19 |
| 56 | *FAM180A* |  | 7q33 | 0.510 | 1.09e-21 | 3.90e-19 |
| 57 | *CLMP* |  | 11q24.1 | 0.509 | 1.18e-21 | 4.14e-19 |
| 58 | *C1QTNF3* |  | 5p13.2 | 0.509 | 1.19e-21 | 4.14e-19 |
| 59 | *LOXL1* |  | 15q24.1 | 0.508 | 1.42e-21 | 4.85e-19 |
| 60 | *COL6A1* |  | 21q22.3 | 0.508 | 1.61e-21 | 5.40e-19 |
| 61 | *ETV1* |  | 7p21.2 | 0.508 | 1.65e-21 | 5.41e-19 |
| 62 | ***SFRP4*** | 32 | 7p14.1 | 0.508 | 1.67e-21 | 5.41e-19 |
| 63 | ***CRISPLD2*** | 33 | 16q24.1 | 0.507 | 1.71e-21 | 5.46e-19 |
| 64 | *SPON2* |  | 4p16.3 | 0.507 | 1.78e-21 | 5.60e-19 |
| 65 | *COL6A2* |  | 21q22.3 | 0.507 | 1.83e-21 | 5.66e-19 |
| 66 | *ECM1* |  | 1q21.2 | 0.507 | 1.95e-21 | 5.95e-19 |
| 67 | *ZNF469* |  | 16q24.2 | 0.505 | 2.58e-21 | 7.75e-19 |
| 68 | ***SFRP2*** | 34 | 4q31.3 | 0.504 | 3.54e-21 | 1.05e-18 |
| 69 | ***CXCL14*** | 35 | 5q31.1 | 0.503 | 3.96e-21 | 1.15e-18 |
| 70 | *CAVIN1* |  | 17q21.2 | 0.502 | 4.93e-21 | 1.42e-18 |
| 71 | *RTL3* |  | Xq21.1 | 0.501 | 6.73e-21 | 1.91e-18 |

**Table S2**. Characteristics of the proteins from physical interaction network generated by STRING v11.5 for MFAP5 and co-expressed proteins.

| **No** | **Gene symbol** | **Function** |
| --- | --- | --- |
| 1 | MFAP5 | Microfibrillar-associated protein 5; May play a role in hematopoiesis. In the cardiovascular system, could regulate growth factors or participate in cell signaling in maintaining large vessel integrity (By similarity). Component of the elastin-associated microfibrils (173 aa) |
| 2 | COL1A2 | Collagen alpha-2(I) chain; Type I collagen is a member of group I collagen (fibrillar forming collagen); Belongs to the fibrillar collagen family (1366 aa) |
| 3 | COL6A3 | Collagen alpha-3(VI) chain; Collagen VI acts as a cell-binding protein; Collagens (3177 aa) |
| 4 | DCN | Decorin; May affect the rate of fibrils formation; Small leucine rich repeat proteoglycans (359 aa) |
| 5 | COL6A2 | Collagen alpha-2(VI) chain; Collagen VI acts as a cell-binding protein; Collagens (1019 aa) |
| 6 | COL3A1 | Collagen alpha-1(III) chain; Collagen type III occurs in most soft connective tissues along with type I collagen. Involved in regulation of cortical development. Is the major ligand of ADGRG1 in the developing brain and binding to ADGRG1 inhibits neuronal migration and activates the RhoA pathway by coupling ADGRG1 to GNA13 and possibly GNA12 (1466 aa) |
| 7 | COL6A1 | Collagen alpha-1(VI) chain; Collagen VI acts as a cell-binding protein; Belongs to the type VI collagen family (1028 aa) |
| 8 | COL1A1 | Collagen alpha-1(I) chain; Type I collagen is a member of group I collagen (fibrillar forming collagen); Collagens (1464 aa) |
| 9 | SPARC | SPARC; Appears to regulate cell growth through interactions with the extracellular matrix and cytokines. Binds calcium and copper, several types of collagen, albumin, thrombospondin, PDGF and cell membranes. There are two calcium binding sites; an acidic domain that binds 5 to 8 Ca(2+) with a low affinity and an EF-hand loop that binds a Ca(2+) ion with a high affinity; SPARC family (303 aa) |
| 10 | THBS2 | Thrombospondin-2; Adhesive glycoprotein that mediates cell-to-cell and cell-to-matrix interactions. Ligand for CD36 mediating antiangiogenic properties (1172 aa) |
| 11 | COL11A1 | Collagen alpha-1(XI) chain; May play an important role in fibrillogenesis by controlling lateral growth of collagen II fibrils; Collagens (1806 aa) |
| 12 | COL5A1 | Collagen alpha-1(V) chain; Type V collagen is a member of group I collagen (fibrillar forming collagen). It is a minor connective tissue component of nearly ubiquitous distribution. Type V collagen binds to DNA, heparan sulfate, thrombospondin, heparin, and insulin; Collagens (1838 aa) |
| 13 | ITGA11 | Integrin alpha-11; Integrin alpha-11/beta-1 is a receptor for collagen (1188 aa) |
| 14 | COL5A2 | Collagen alpha-2(V) chain; Type V collagen is a member of group I collagen (fibrillar forming collagen). It is a minor connective tissue component of nearly ubiquitous distribution. Type V collagen binds to DNA, heparan sulfate, thrombospondin, heparin, and insulin. Type V collagen is a key determinant in the assembly of tissue- specific matrices (By similarity) (1499 aa) |
| 15 | TIMP3 | Metalloproteinase inhibitor 3; Complexes with metalloproteinases (such as collagenases) and irreversibly inactivates them by binding to their catalytic zinc cofactor. May form part of a tissue-specific acute response to remodeling stimuli. Known to act on MMP-1, MMP-2, MMP-3, MMP-7, MMP-9, MMP-13, MMP-14 and MMP-15 (211 aa) |
| 16 | FBN1 | Fibrillin-1; Fibrillin-1: Structural component of the 10-12 nm diameter microfibrils of the extracellular matrix, which conveys both structural and regulatory properties to load-bearing connective tissues. Fibrillin-1- containing microfibrils provide long-term force bearing structural support. In tissues such as the lung, blood vessels and skin, microfibrils form the periphery of the elastic fiber, acting as a scaffold for the deposition of elastin. In addition, microfibrils can occur as elastin-independent networks in tissues such as the ciliary zonule, tendon, cornea and glomerulus w [...] (2871 aa) |
| 17 | CALD1 | Caldesmon; Actin- and myosin-binding protein implicated in the regulation of actomyosin interactions in smooth muscle and nonmuscle cells (could act as a bridge between myosin and actin filaments). Stimulates actin binding of tropomyosin which increases the stabilization of actin filament structure. In muscle tissues, inhibits the actomyosin ATPase by binding to F-actin. This inhibition is attenuated by calcium-calmodulin and is potentiated by tropomyosin. Interacts with actin, myosin, two molecules of tropomyosin and with calmodulin. Also play an essential role during cellular mitosis [...] (793 aa) |
| 18 | VCAN | Versican core protein; May play a role in intercellular signaling and in connecting cells with the extracellular matrix. May take part in the regulation of cell motility, growth and differentiation. Binds hyaluronic acid; C-type lectin domain containing (3396 aa) |
| 19 | WISP1 | WNT1-inducible-signaling pathway protein 1; Downstream regulator in the Wnt/Frizzled-signaling pathway. Associated with cell survival. Attenuates p53-mediated apoptosis in response to DNA damage through activation of AKT kinase. Up-regulates the anti-apoptotic Bcl-X(L) protein. Adheres to skin and melanoma fibroblasts. In vitro binding to skin fibroblasts occurs through the proteoglycans, decorin and biglycan; Belongs to the CCN family (367 aa) |
| 20 | MMP2 | 72 kDa type IV collagenase; Ubiquitinous metalloproteinase that is involved in diverse functions such as remodeling of the vasculature, angiogenesis, tissue repair, tumor invasion, inflammation, and atherosclerotic plaque rupture. As well as degrading extracellular matrix proteins, can also act on several nonmatrix proteins such as big endothelial 1 and beta-type CGRP promoting vasoconstriction. Also cleaves KISS at a Gly-\|-Leu bond. Appears to have a role in myocardial cell death pathways. Contributes to myocardial oxidative stress by regulating the activity of GSK3beta. Cleaves GSK3b [...] (660 aa) |
| 21 | LUM | Lumican; Small leucine rich repeat proteoglycans; Belongs to the small leucine-rich proteoglycan (SLRP) family. SLRP class II subfamily (338 aa) |
| 22 | ACTA2 | Actin, aortic smooth muscle; Actins are highly conserved proteins that are involved in various types of cell motility and are ubiquitously expressed in all eukaryotic cells; Belongs to the actin family (377 aa) |


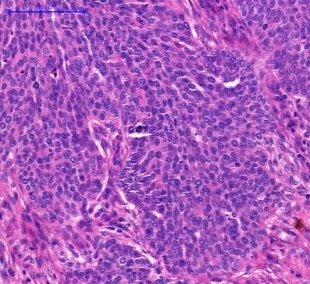

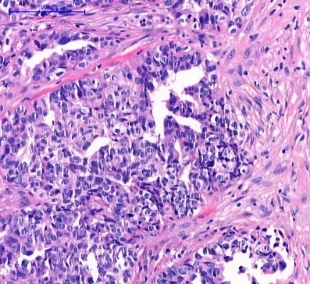

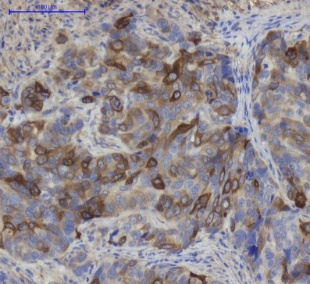

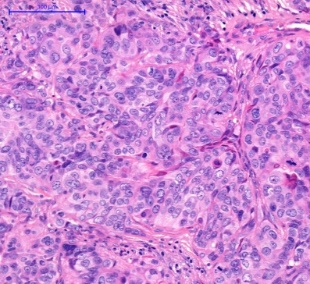

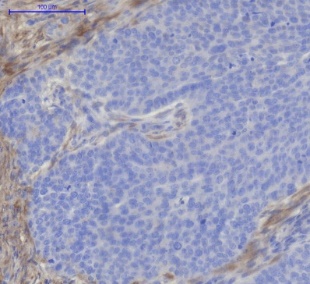

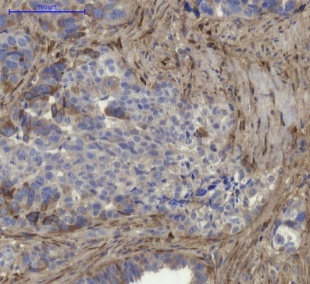


**MFAP5 expression in cancer cells**

**Score 2**

**Score 1**

**Score 3**

H&E

**MFAP5 expression in tumor stroma**

**Score 2**

**Score 1**

**Score 3**

H&E


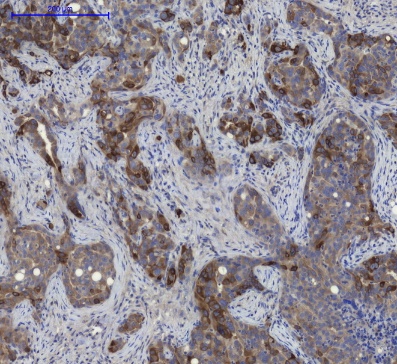

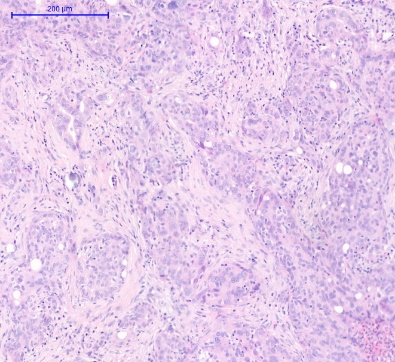

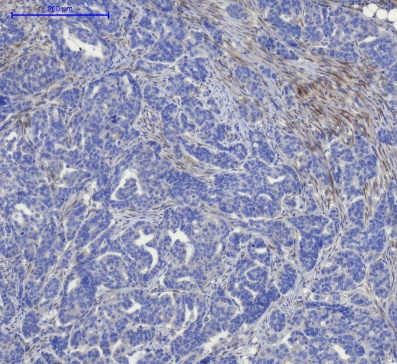

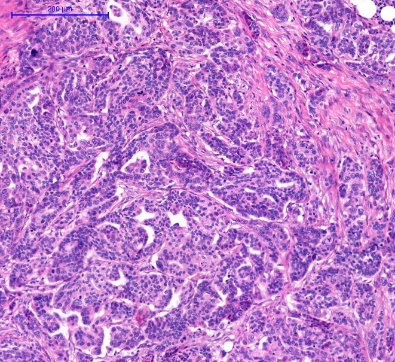

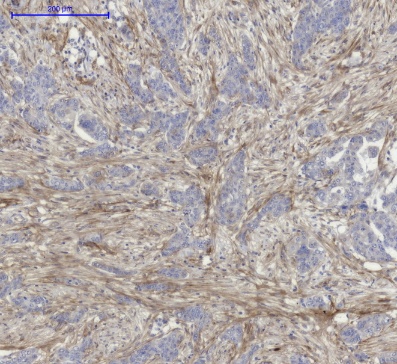

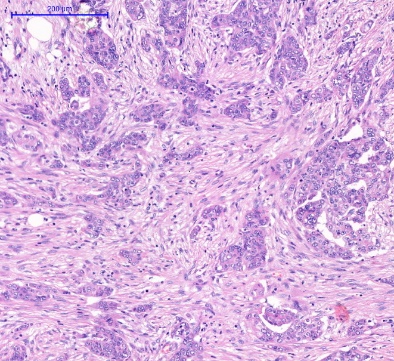


**Figure S2.** Immunohistochemical detection of MFAP5 protein in ovarian cancer samples. Upper panel - representative examples of MFAP5 staining in cancer cells and corresponding H&E staining. Lower panel - representative examples of MFAP5 staining in the tumor stroma and corresponding H&E staining. The images show different levels of staining, from score 1 (weak expression), through score 2 (moderate expression), to score 3 (strong expression). Pannoramic 250 Flash II Scanner, scale bar: 100 µm.

**Table S3.** The association between MFAP5 protein expression in the tumor and clinico-pathological features (exact Fisher test, p<0.05).

| Characteristics | Number of patients | | MFAP5 expression in cancer cells | | | | P value | MFAP5 expression in tumor stroma | | | | P value |
| --- | --- | --- | --- | --- | --- | --- | --- | --- | --- | --- | --- | --- |
|  |  |  | Weak  (n=67, 62%) | | Moderate & strong  (n=41, 38%) | |  | Weak  (n=81, 75%) | | Moderate & strong  (n=27, 25%) | |  |
|  | n | % | n | % | n | % |  | n | % | n | % |  |
| Age |  |  |  |  |  |  |  |  |  |  |  |  |
| ≤ 54 years | 56 | 51.85 | 37 | 66.07 | 19 | 33.93 | 0.430 | 40 | 71.43 | 16 | 28.57 | 0.505 |
| > 54 years | 52 | 48.15 | 30 | 57.69 | 22 | 42.31 |  | 41 | 78.85 | 11 | 21.15 |  |
|  |  |  |  |  |  |  |  |  |  |  |  |  |
| Resiudal disease |  |  |  |  |  |  |  |  |  |  |  |  |
| No | 17 | 15.74 | 10 | 58.82 | 7 | 41.18 | 0.790 | 12 | 70.59 | 5 | 29.41 | 0.761 |
| Yes | 91 | 84.26 | 57 | 62.64 | 34 | 37.36 |  | 69 | 75.82 | 22 | 24.18 |  |
|  |  |  |  |  |  |  |  |  |  |  |  |  |
| Grade* |  |  |  |  |  |  |  |  |  |  |  |  |
| G3 | 76 | 71.70 | 44 | 57.89 | 32 | 42.11 | 0.277 | 61 | 80.26 | 15 | 19.74 | **0.082** |
| G4 | 30 | 28.30 | 21 | 70.00 | 9 | 30.00 |  | 19 | 63.33 | 11 | 36.67 |  |
|  |  |  |  |  |  |  |  |  |  |  |  |  |
| Platinum sensitivity |  |  |  |  |  |  |  |  |  |  |  |  |
| Highly sensitive (DFS > 24 mths) | 22 | 20.37 | 12 | 54.55 | 10 | 45.45 | 0.736 | 17 | 77.27 | 5 | 22.73 | 0.919 |
| Moderately sensitive (24 mths > DFS > 6 mths) | 42 | 38.89 | 27 | 64.29 | 15 | 35.71 |  | 32 | 76.19 | 10 | 23.81 |  |
| Resistant (DFS < 6 mths) | 44 | 40.74 | 28 | 63.64 | 16 | 36.36 |  | 32 | 72.73 | 12 | 27.27 |  |
|  |  |  |  |  |  |  |  |  |  |  |  |  |
| TP53 accumulation |  |  |  |  |  |  |  |  |  |  |  |  |
| - | 40 | 37.04 | 27 | 67.50 | 13 | 32.50 | 0.416 | 33 | 82.50 | 7 | 17.50 | 0.249 |
| + | 68 | 62.96 | 40 | 58.82 | 28 | 41.18 |  | 48 | 70.59 | 20 | 29.41 |  |
|  |  |  |  |  |  |  |  |  |  |  |  |  |
| CHT response |  |  |  |  |  |  |  |  |  |  |  |  |
| CR | 74 | 68.52 | 46 | 62.16 | 28 | 37.84 | **0.079** | 57 | 77.03 | 17 | 22.97 | 0.432 |
| PR | 31 | 28.70 | 21 | 67.74 | 10 | 32.26 |  | 21 | 67.74 | 10 | 32.26 |  |
| NC or P | 3 | 2.78 | 0 | 0.00 | 3 | 100.00 |  | 3 | 100.00 | 0 | 0.00 |  |
|  |  |  |  |  |  |  |  |  |  |  |  |  |
| Growth type |  |  |  |  |  |  |  |  |  |  |  |  |
| Papillary | 17 | 15.74 | 12 | 70.59 | 5 | 29.41 | 0.796 | 14 | 82.35 | 3 | 17.65 | 0.471 |
| Solid | 34 | 31.48 | 21 | 61.76 | 13 | 38.24 |  | 23 | 67.65 | 11 | 32.35 |  |
| Mixed | 57 | 52.78 | 34 | 59.65 | 23 | 40.35 |  | 44 | 77.19 | 13 | 22.81 |  |
|  |  |  |  |  |  |  |  |  |  |  |  |  |
| Mitotic activity |  |  |  |  |  |  |  |  |  |  |  |  |
| 1 (0-9/10HPF) | 11 | 10.19 | 6 | 54.55 | 5 | 45.45 | 0.906 | 8 | 72.73 | 3 | 27.27 | 0.615 |
| 2 (10-24/10HPF) | 25 | 23.15 | 16 | 64.00 | 9 | 36.00 |  | 17 | 68.00 | 8 | 32.00 |  |
| 3 (>24/10HPF) | 72 | 66.67 | 45 | 62.50 | 27 | 37.50 |  | 56 | 77.78 | 16 | 22.22 |  |
|  |  |  |  |  |  |  |  |  |  |  |  |  |
| Source of tumor sample |  |  |  |  |  |  |  |  |  |  |  |  |
| Ovary (O) | 39 | 36.11 | 24 | 61.54 | 15 | 38.46 | 0.800 | 33 | 84.62 | 6 | 15.38 | 0.215 |
| Tumor (T) | 33 | 30.56 | 22 | 66.67 | 11 | 33.33 |  | 23 | 69.70 | 10 | 30.30 |  |
| Peritoneum (P) | 36 | 33.33 | 21 | 58.33 | 15 | 41.67 |  | 25 | 69.44 | 11 | 30.56 |  |
|  |  |  |  |  |  |  |  |  |  |  |  |  |
| Inflammatory infiltration |  |  |  |  |  |  |  |  |  |  |  |  |
| Weak | 81 | 75.00 | 51 | 62.96 | 30 | 37.04 | 0.820 | 57 | 70.37 | 24 | 29.63 | **0.072** |
| Moderate & Strong | 27 | 25.00 | 16 | 59.26 | 11 | 40.74 |  | 24 | 88.89 | 3 | 11.11 |  |
|  |  |  |  |  |  |  |  |  |  |  |  |  |
| Degree of desmoplastic reaction |  |  |  |  |  |  |  |  |  |  |  |  |
| 1 | 51 | 47.22 | 38 | 74.51 | 13 | 25.49 | **0.035** | 48 | 94.12 | 3 | 5.88 | **< 0.001** |
| 2 | 47 | 43.52 | 23 | 48.94 | 24 | 51.06 |  | 30 | 63.83 | 17 | 36.17 |  |
| 3 | 10 | 9.26 | 6 | 60.00 | 4 | 40.00 |  | 3 | 30.00 | 7 | 70.00 |  |
|  |  |  |  |  |  |  |  |  |  |  |  |  |
| Necrosis |  |  |  |  |  |  |  |  |  |  |  |  |
| - | 28 | 25.93 | 17 | 60.71 | 11 | 39.29 | 1.00 | 19 | 67.86 | 9 | 32.14 | 0.321 |
| + | 80 | 74.07 | 50 | 62.50 | 30 | 37.50 |  | 62 | 77.50 | 18 | 22.50 |  |
|  |  |  |  |  |  |  |  |  |  |  |  |  |
| Calcification |  |  |  |  |  |  |  |  |  |  |  |  |
| - | 78 | 72.22 | 50 | 64.10 | 28 | 35.90 | 0.512 | 58 | 74.36 | 20 | 25.64 | 1.00 |
| + | 30 | 27.78 | 17 | 56.67 | 13 | 43.33 |  | 23 | 76.67 | 7 | 23.33 |  |
|  |  |  |  |  |  |  |  |  |  |  |  |  |
| Angioinvasion |  |  |  |  |  |  |  |  |  |  |  |  |
| No | 23 | 21.30 | 15 | 65.22 | 8 | 34.78 | 0.812 | 20 | 86.96 | 3 | 13.04 | 0.179 |
| Yes | 85 | 78.70 | 52 | 61.18 | 33 | 38.82 |  | 61 | 71.76 | 24 | 28.24 |  |
| * G2 ignored, because of only two patients with G2;  HPF – high power field (400-fold magnification);  CHT response: CR – complete response, PR – partial response, NC – no change, P – progression;  O – tumor section containing ovarian structure(s), T – section with tumor tissue only, P – section containing peritoneal/omental structures;  Stromal MFAP5 expression: weak – score 1, moderate – score 2, strong – score 3 | | | | | | | | | | | | |

**MFAP5 in cancer cells**

log-rank test, p=0.761

log-rank test, p=0.238

log-rank test, p=0.34

log-rank test, p=0.592

**MFAP5 in tumor stroma**

**Overall survival**

**Disease-free survival**

**Figure S3.** Kaplan-Meier analysis of overall survival (OS) and disease-free survival (DFS) in 108 patients with HG-SOC, stratified by stronger MFAP5 protein expression (IHC score 2 & 3) versus weak MFAP5 expression (IHC score 1) in cancer cells (upper panel) and the tumor stroma (lower panel).

**Characteristic of tissue arrays**

T112b


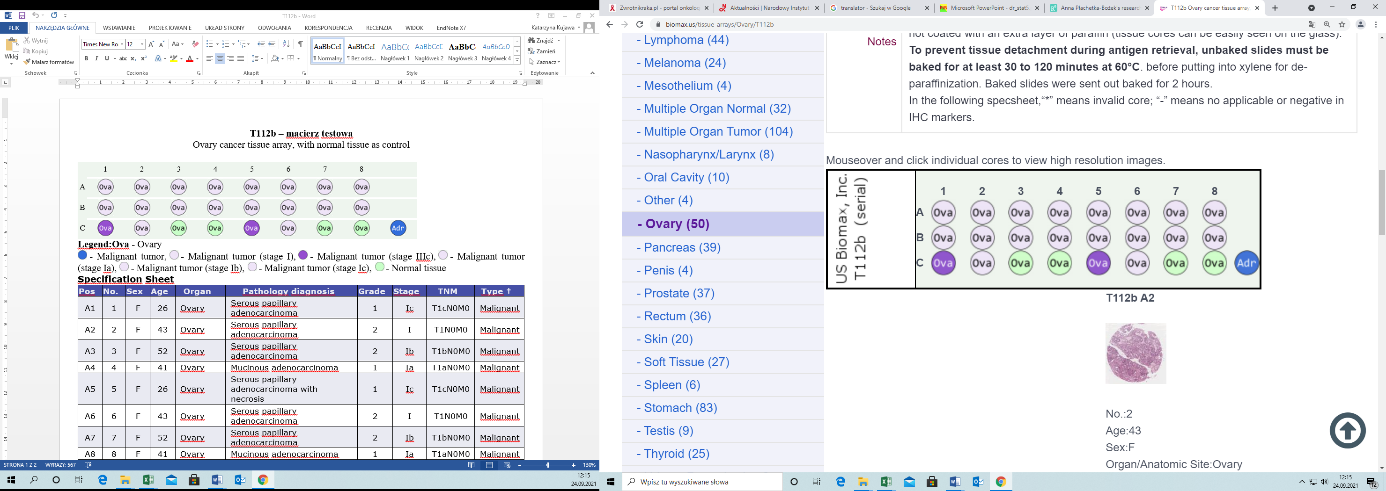


**Legend: Ova** - Ovary

 - Malignant tumor, 
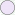
 - Malignant tumor (stage I), 
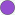
 - Malignant tumor (stage IIIc), 
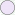
 - Malignant tumor (stage Ia), 
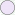
 - Malignant tumor (stage Ib), 
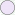
 - Malignant tumor (stage Ic), 
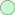
 - Normal tissue


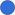


**Figure S4.** Ovary cancer tissue T112b, US Biomax.

**Table S4.** US Biomax, T112b.

| [**Pos**](http://www.biomax.us/) | [**No.**](http://www.biomax.us/) | [**Sex**](http://www.biomax.us/) | [**Age**](http://www.biomax.us/) | [**Organ**](http://www.biomax.us/) | [**Pathology diagnosis**](http://www.biomax.us/) | [**Grade**](http://www.biomax.us/) | [**Stage**](http://www.biomax.us/) | [**TNM**](http://www.biomax.us/) | [**Type †**](http://www.biomax.us/) |
| --- | --- | --- | --- | --- | --- | --- | --- | --- | --- |
| A1 | 1 | F | 26 | Ovary | Serous papillary adenocarcinoma | 1 | Ic | T1cN0M0 | Malignant |
| A2 | 2 | F | 43 | Ovary | Serous papillary adenocarcinoma | 2 | I | T1N0M0 | Malignant |
| A3 | 3 | F | 52 | Ovary | Serous papillary adenocarcinoma | 2 | Ib | T1bN0M0 | Malignant |
| A4 | 4 | F | 41 | Ovary | Mucinous adenocarcinoma | 1 | Ia | T1aN0M0 | Malignant |
| A5 | 5 | F | 26 | Ovary | Serous papillary adenocarcinoma with necrosis | 1 | Ic | T1cN0M0 | Malignant |
| A6 | 6 | F | 43 | Ovary | Serous papillary adenocarcinoma | 2 | I | T1N0M0 | Malignant |
| A7 | 7 | F | 52 | Ovary | Serous papillary adenocarcinoma | 2 | Ib | T1bN0M0 | Malignant |
| A8 | 8 | F | 41 | Ovary | Mucinous adenocarcinoma | 1 | Ia | T1aN0M0 | Malignant |
| B1 | 9 | F | 42 | Ovary | Mucinous adenocarcinoma | 1–2 | Ib | T1bN0M0 | Malignant |
| B2 | 10 | F | 49 | Ovary | Clear cell carcinoma | – | I | T1N0M0 | Malignant |
| B3 | 11 | F | 37 | Ovary | Clear cell carcinoma | – | Ia | T1aN0M0 | Malignant |
| B4 | 12 | F | 43 | Ovary | Endometrioid adenocarcinoma | 1–2 | Ib | T1bN0M0 | Malignant |
| B5 | 13 | F | 42 | Ovary | Mucinous adenocarcinoma | 1–2 | Ib | T1bN0M0 | Malignant |
| B6 | 14 | F | 49 | Ovary | Clear cell carcinoma | – | I | T1N0M0 | Malignant |
| B7 | 15 | F | 37 | Ovary | Clear cell carcinoma | – | Ia | T1aN0M0 | Malignant |
| B8 | 16 | F | 43 | Ovary | Endometrioid adenocarcinoma with necrosis | 1–2 | Ib | T1bN0M0 | Malignant |
| C1 | 17 | F | 40 | Ovary | Endometrioid adenocarcinoma | 2 | IIIc | T2N1M0 | Malignant |
| C2 | 18 | F | 25 | Ovary | Granular cell tumor | – | I | T1N0M0 | Malignant |
| C3 | 19 | F | 34 | Ovary | Ovary tissue | – | – | – | Normal |
| C4 | 20 | F | 19 | Ovary | Ovary tissue | – | – | – | Normal |
| C5 | 21 | F | 40 | Ovary | Endometrioid adenocarcinoma | 2 | IIIc | T2N1M0 | Malignant |
| C6 | 22 | F | 25 | Ovary | Granular cell tumor | – | I | T1N0M0 | Malignant |
| C7 | 23 | F | 34 | Ovary | Ovary tissue | – | – | – | Normal |
| C8 | 24 | F | 19 | Ovary | Ovary tissue | – | – | – | Normal |
| – | – | M | 42 | Adrenal gland | Pheochromocytoma (tissue marker) | – |  |  | Malignant |

OV1005a


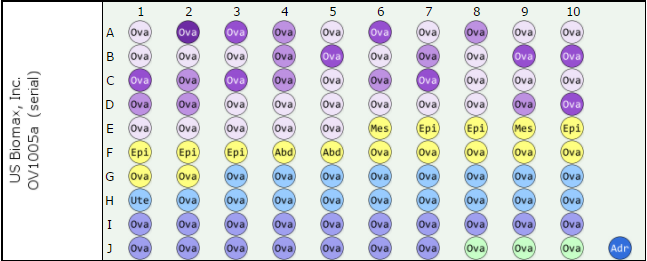


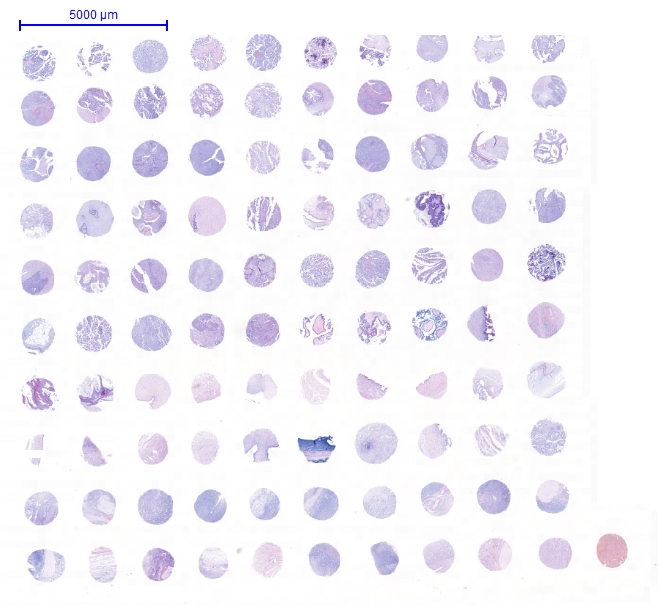


**H+E OV1005a** **tissue array**

| **Legend:** | **Abd** - Abdominal cavity, **Epi** - Epiploon, **Mes** - Mesentery, **Ova** - Ovary, **Ute** - Uterus   - Benign tumor, 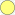 - Borderline, 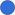 - Malignant tumor, 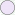 - Malignant tumor (stage I), 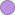 - Malignant tumor (stage II), 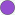 - Malignant tumor (stage IIIc), 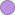 - Malignant tumor (stage IIa), 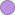 - Malignant tumor (stage IIb), 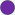 - Malignant tumor (stage IV), 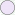 - Malignant tumor (stage Ia), 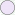 - Malignant tumor (stage Ib), 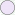 - Malignant tumor (stage Ic), 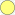 - Metastasis, 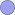 - NAT, 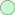 - Normal tissue 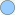 |
| --- | --- |

**Figure S5.** “Ovary disease spectrum (ovarian cancer progression) tissue array” OV1005a, US Biomax.

**Table S5.** US Biomax, OV1005a.

| [**Pos**](http://www.biomax.us/) | [**No.**](http://www.biomax.us/) | [**Sex**](http://www.biomax.us/) | [**Age**](http://www.biomax.us/) | [**Organ**](http://www.biomax.us/) | [**Pathology diagnosis**](http://www.biomax.us/) | [**Grade**](http://www.biomax.us/) | [**Stage**](http://www.biomax.us/) | [**TNM**](http://www.biomax.us/) | [**Type †**](http://www.biomax.us/) |
| --- | --- | --- | --- | --- | --- | --- | --- | --- | --- |
| A1 | 1 | F | 65 | Ovary | Serous papillary cystadenocarcinoma | 1 | I | T1N0M0 | Malignant |
| A2 | 2 | F | 38 | Ovary | Serous papillary cystadenocarcinoma | 1 | IV | T3cN1M1 | Malignant |
| A3 | 3 | F | 51 | Ovary | Serous papillary cystadenocarcinoma | 1 | IIIc | T3cN1M0 | Malignant |
| A4 | 4 | F | 22 | Ovary | Serous papillary cystadenocarcinoma | 1 | IIb | T2bN0M0 | Malignant |
| A5 | 5 | F | 48 | Ovary | Serous papillary cystadenocarcinoma | 1 | I | T1N0M0 | Malignant |
| A6 | 6 | F | 26 | Ovary | Serous papillary cystadenocarcinoma | 1 | IIIc | T3cN1M0 | Malignant |
| A7 | 7 | F | 25 | Ovary | Serous papillary cystadenocarcinoma | 1 | I | T1N0M0 | Malignant |
| A8 | 8 | F | 50 | Ovary | Serous papillary cystadenocarcinoma | 1 | II | T2N0M0 | Malignant |
| A9 | 9 | F | 26 | Ovary | Serous papillary cystadenocarcinoma | 1 | Ic | T1cN0M0 | Malignant |
| A10 | 10 | F | 47 | Ovary | Serous papillary cystadenocarcinoma | 1 | I | T1N0M0 | Malignant |
| B1 | 11 | F | 58 | Ovary | Serous papillary adenocarcinoma with necrosis | 2 | I | T1N0M0 | Malignant |
| B2 | 12 | F | 57 | Ovary | Serous papillary cystadenocarcinoma | 2 | Ic | T1cN0M0 | Malignant |
| B3 | 13 | F | 51 | Ovary | Serous papillary adenocarcinoma | 2 | Ia | T1aN0M0 | Malignant |
| B4 | 14 | F | 52 | Ovary | Serous papillary cystadenocarcinoma | 2 | II | T2N0M0 | Malignant |
| B5 | 15 | F | 54 | Ovary | Serous papillary adenocarcinoma | 2 | IIIc | T3cN1M0 | Malignant |
| B6 | 16 | F | 33 | Ovary | Serous papillary adenocarcinoma | 2 | I | T1N0M0 | Malignant |
| B7 | 17 | F | 56 | Ovary | Serous papillary adenocarcinoma | 3 | II | T2N0M0 | Malignant |
| B8 | 18 | F | 41 | Ovary | Serous papillary adenocarcinoma | 2 | I | T1N0M0 | Malignant |
| B9 | 19 | F | 46 | Ovary | Serous papillary adenocarcinoma | 2 | IIIc | T3aN0M0 | Malignant |
| B10 | 20 | F | 46 | Ovary | Serous papillary adenocarcinoma | 2 | IIIc | T2cN1M0 | Malignant |
| C1 | 21 | F | 57 | Ovary | Serous adenocarcinoma | 2 | IIIc | T3cN1M0 | Malignant |
| C2 | 22 | F | 75 | Ovary | Serous adenocarcinoma | 2–3 | II | T2N0M0 | Malignant |
| C3 | 23 | F | 54 | Ovary | Serous adenocarcinoma | 3 | IIIc | T3cN1M0 | Malignant |
| C4 | 24 | F | 49 | Ovary | Serous adenocarcinoma | 3 | II | T2N0M0 | Malignant |
| C5 | 25 | F | 50 | Ovary | Serous papillary adenocarcinoma | 2 | I | T1N0M0 | Malignant |
| C6 | 26 | F | 52 | Ovary | Serous adenocarcinoma | 3 | II | T2N0M0 | Malignant |
| C7 | 27 | F | 47 | Ovary | Serous adenocarcinoma | 3 | IIIc | T3cN1M0 | Malignant |
| C8 | 28 | F | 34 | Ovary | Mucinous adenocarcinoma | 2 | Ib | T1bN0M0 | Malignant |
| C9 | 29 | F | 63 | Ovary | Mucinous adenocarcinoma | 1 | Ia | T1aN0M0 | Malignant |
| C10 | 30 | F | 69 | Ovary | Mucinous adenocarcinoma | 1 | Ib | T1bN0M0 | Malignant |
| D1 | 31 | F | 46 | Ovary | Endometrioid adenocarcinoma | 1–2 | II | T2N0M0 | Malignant |
| D2 | 32 | F | 47 | Ovary | Endometrioid adenocarcinoma | 1–2 | IIa | T2aN0M0 | Malignant |
| D3 | 33 | F | 54 | Ovary | Endometrioid adenocarcinoma | 1–2 | Ib | T1bN0M0 | Malignant |
| D4 | 34 | F | 65 | Ovary | Adenocarcinoma (sparse) | – | Ic | T1cN0M0 | Malignant |
| D5 | 35 | F | 55 | Ovary | Endometrioid adenocarcinoma | 2 | I | T1N0M0 | Malignant |
| D6 | 36 | F | 54 | Ovary | Endometrioid adenocarcinoma | 1 | Ib | T1bN0M0 | Malignant |
| D7 | 37 | F | 43 | Ovary | Endometrioid adenocarcinoma | 1 | Ic | T1cN0M0 | Malignant |
| D8 | 38 | F | 55 | Ovary | Endometrioid adenocarcinoma with necrosis | 1 | I | T1N0M0 | Malignant |
| D9 | 39 | F | 53 | Ovary | Endometrioid adenocarcinoma | 3 | IIa | T2aN0M0 | Malignant |
| D10 | 40 | F | 50 | Ovary | Endometrioid adenocarcinoma | 2 | IIIc | T3bN1M0 | Malignant |
| E1 | 41 | F | 51 | Ovary | Transitional cell carcinoma with necrosis | 2 | Ib | T1bN0M0 | Malignant |
| E2 | 42 | F | 39 | Ovary | Transitional cell carcinoma with necrosis | 2 | Ia | T1aN0M0 | Malignant |
| E3 | 43 | F | 38 | Ovary | Transitional cell carcinoma | 2–3 | I | T1N0M0 | Malignant |
| E4 | 44 | F | 66 | Ovary | Transitional cell carcinoma with squamous metaplasia | 3 | Ia | T1aN0M0 | Malignant |
| E5 | 45 | F | 53 | Ovary | Transitional cell carcinoma | 2–3 | I | T1N0M0 | Malignant |
| E6 | 46 | F | 47 | Mesentery | Metastatic serous papillary cystadenocarcinoma from ovary | 1 | – | – | Metastasis |
| E7 | 47 | F | 57 | Epiploon | Metastatic serous papillary cystadenocarcinoma from ovary | 1 | – | – | Metastasis |
| E8 | 48 | F | 65 | Epiploon | Metastatic serous papillary cystadenocarcinoma with calcification from ovary | 1 | – | – | Metastasis |
| E9 | 49 | F | 59 | Mesentery | Metastatic serous papillary cystadenocarcinoma from ovary | 2 | – | – | Metastasis |
| E10 | 50 | F | 28 | Epiploon | Metastatic serous adenocarcinoma with calcification from ovary | 2 | – | – | Metastasis |
| F1 | 51 | F | 64 | Epiploon | Metastatic serous papillary cystadenocarcinoma from ovary | 1 | – | – | Metastasis |
| F2 | 52 | F | 50 | Epiploon | Metastatic serous papillary cystadenocarcinoma from ovary | 1 | – | – | Metastasis |
| F3 | 53 | F | 58 | Epiploon | Metastatic adenocarcinoma from ovary | 2 | – | – | Metastasis |
| F4 | 54 | F | 47 | Abdominal cavity | Metastatic adenocarcinoma from ovary | 2 | – | – | Metastasis |
| F5 | 55 | F | 49 | Abdominal cavity | Metastatic adenocarcinoma from ovary | 3 | – | – | Metastasis |
| F6 | 56 | F | 34 | Ovary | Borderline serous papillary cystadenoma | – | – | – | Borderline |
| F7 | 57 | F | 34 | Ovary | Borderline serous papillary cystadenoma | – | – | – | Borderline |
| F8 | 58 | F | 28 | Ovary | Borderline serous papillary cystadenoma | – | – | – | Borderline |
| F9 | 59 | F | 22 | Ovary | Borderline serous papillary cystadenoma | – | – | – | Borderline |
| F10 | 60 | F | 60 | Ovary | Borderline serous papillary cystadenoma | – | – | – | Borderline |
| G1 | 61 | F | 50 | Ovary | Borderline serous papillary cystadenoma | – | – | – | Borderline |
| G2 | 62 | F | 37 | Ovary | Borderline mucinous papillary cystadenoma | – | – | – | Borderline |
| G3 | 63 | F | 62 | Ovary | Serous cystadenoma | – | – | – | Benign |
| G4 | 64 | F | 70 | Ovary | Serous cystadenoma | – | – | – | Benign |
| G5 | 65 | F | 49 | Ovary | Serous cystadenoma | – | – | – | Benign |
| G6 | 66 | F | 16 | Ovary | Serous cystadenoma | – | – | – | Benign |
| G7 | 67 | F | 34 | Ovary | Serous cystadenoma | – | – | – | Benign |
| G8 | 68 | F | 22 | Ovary | Serous cystadenoma | – | – | – | Benign |
| G9 | 69 | F | 19 | Ovary | Mucinous cystadenoma | – | – | – | Benign |
| G10 | 70 | F | 17 | Ovary | Mucinous cystadenoma | – | – | – | Benign |
| H1 | 71 | F | 41 | Uterus | Mucinous cystadenoma (ovary tissue) | – | – | – | Benign |
| H2 | 72 | F | 26 | Ovary | Mucinous cystadenoma | – | – | – | Benign |
| H3 | 73 | F | 22 | Ovary | Mucinous cystadenoma | – | – | – | Benign |
| H4 | 74 | F | 38 | Ovary | Mucinous cystadenoma | – | – | – | Benign |
| H5 | 75 | F | 47 | Ovary | Mucinous cystadenoma | – | – | – | Benign |
| H6 | 76 | F | 70 | Ovary | Mucinous cystadenoma | – | – | – | Benign |
| H7 | 77 | F | 51 | Ovary | Mucinous cystadenoma | – | – | – | Benign |
| H8 | 78 | F | 29 | Ovary | Mucinous cystadenoma (fibrous tissue and blood vessel) | – | – | – | Benign |
| H9 | 79 | F | 35 | Ovary | Mucinous cystadenoma (ovary tissue) | – | – | – | Benign |
| H10 | 80 | F | 18 | Ovary | Mucinous cystadenoma (fibrous tissue and blood vessel) | – | – | – | Benign |
| I1 | 81 | F | 30 | Ovary | Cancer adjacent normal ovary tissue | – | – | – | NAT |
| I2 | 82 | F | 39 | Ovary | Cancer adjacent normal ovary tissue | – | – | – | NAT |
| I3 | 83 | F | 29 | Ovary | Cancer adjacent normal ovary tissue | – | – | – | NAT |
| I4 | 84 | F | 41 | Ovary | Cancer adjacent normal ovary tissue | – | – | – | NAT |
| I5 | 85 | F | 62 | Ovary | Cancer adjacent normal ovary tissue | – | – | – | NAT |
| I6 | 86 | F | 63 | Ovary | Cancer adjacent normal ovary tissue | – | – | – | NAT |
| I7 | 87 | F | 45 | Ovary | Cancer adjacent normal ovary tissue | – | – | – | NAT |
| I8 | 88 | F | 48 | Ovary | Cancer adjacent normal ovary tissue | – | – | – | NAT |
| I9 | 89 | F | 53 | Ovary | Cancer adjacent normal ovary tissue | – | – | – | NAT |
| I10 | 90 | F | 53 | Ovary | Cancer adjacent normal ovary tissue | – | – | – | NAT |
| J1 | 91 | F | 57 | Ovary | Cancer adjacent normal ovary tissue | – | – | – | NAT |
| J2 | 92 | F | 38 | Ovary | Cancer adjacent normal ovary tissue | – | – | – | NAT |
| J3 | 93 | F | 53 | Ovary | Cancer adjacent normal ovary tissue | – | – | – | NAT |
| J4 | 94 | F | 59 | Ovary | Cancer adjacent normal ovary tissue | – | – | – | NAT |
| J5 | 95 | F | 48 | Ovary | Cancer adjacent normal ovary tissue | – | – | – | NAT |
| J6 | 96 | F | 50 | Ovary | Cancer adjacent normal ovary tissue | – | – | – | NAT |
| J7 | 97 | F | 52 | Ovary | Cancer adjacent normal ovary tissue | – | – | – | NAT |
| J8 | 98 | F | 27 | Ovary | Normal ovary tissue | – | – | – | Normal |
| J9 | 99 | F | 34 | Ovary | Normal ovary tissue | – | – | – | Normal |
| J10 | 100 | F | 19 | Ovary | Normal ovary tissue | – | – | – | Normal |
| – | – | M | 42 | Adrenal gland | Pheochromocytoma (tissue marker) | – |  |  | Malignant |

**BC11115c**


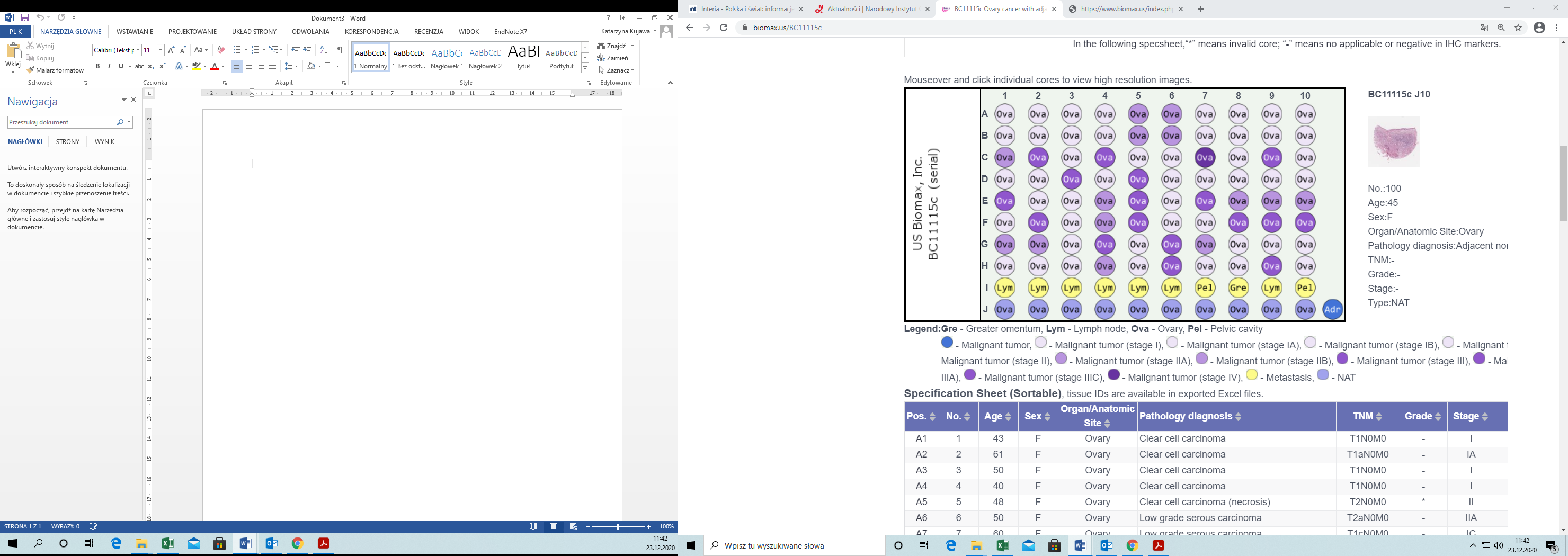

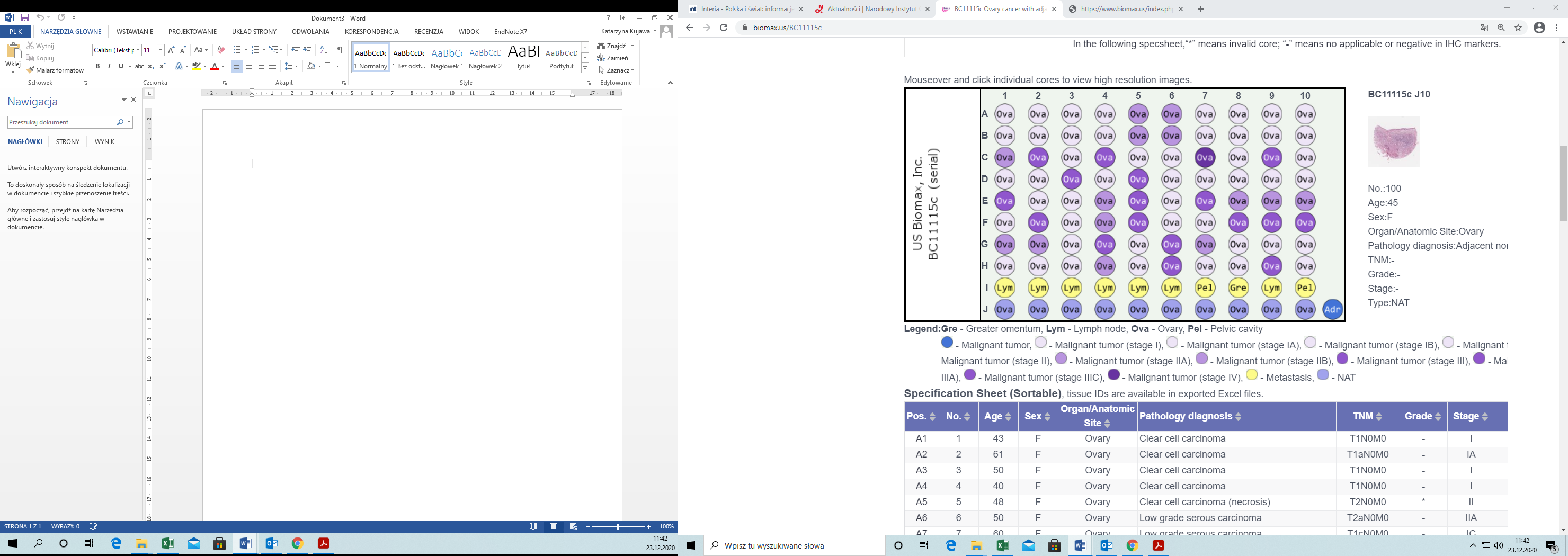

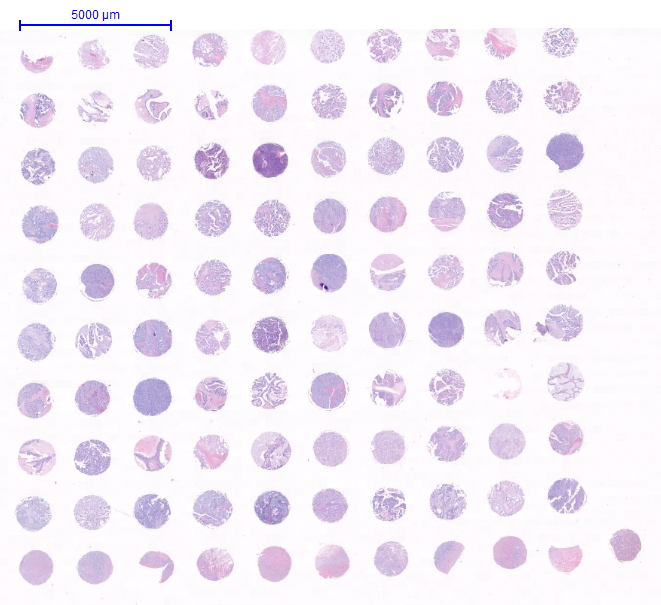


**H+E BC11115c tissue array**

**Figure S6.** Tissue array with spectrum of different histological types of ovarian cancer BC11115c, US Biomax.

**Table S6.** US Biomax, BC11115c.

| Position | No. | Age | Organ/Anatomic Site | Pathology diagnosis | TNM | Grade | Stage | Type |
| --- | --- | --- | --- | --- | --- | --- | --- | --- |
|  |  |  |  |  |  |  |  |  |
| A1 | 1 | 43 | Ovary | Clear cell carcinoma | T1N0M0 | - | I | Malignant |
| A2 | 2 | 61 | Ovary | Clear cell carcinoma | T1aN0M0 | - | IA | Malignant |
| A3 | 3 | 50 | Ovary | Clear cell carcinoma | T1N0M0 | - | I | Malignant |
| A4 | 4 | 40 | Ovary | Clear cell carcinoma | T1N0M0 | - | I | Malignant |
| A5 | 5 | 48 | Ovary | Clear cell carcinoma (necrosis) | T2N0M0 | * | II | Malignant |
| A6 | 6 | 50 | Ovary | Low grade serous carcinoma | T2aN0M0 | - | IIA | Malignant |
| A7 | 7 | 60 | Ovary | Low grade serous carcinoma | T1cN0M0 | - | IC | Malignant |
| A8 | 8 | 69 | Ovary | Endometrioid adenocarcinoma | T1aN0M0 | 2 | IA | Malignant |
| A9 | 9 | 41 | Ovary | Low grade serous carcinoma | T1N0M0 | - | I | Malignant |
| A10 | 10 | 37 | Ovary | Low grade serous carcinoma | T1aN0M0 | - | IA | Malignant |
| B1 | 11 | 25 | Ovary | Low grade serous carcinoma | T1N0M0 | - | I | Malignant |
| B2 | 12 | 34 | Ovary | Low grade serous carcinoma | T1aN0M0 | - | IA | Malignant |
| B3 | 13 | 59 | Ovary | Low grade serous carcinoma | T1aN0M0 | - | IA | Malignant |
| B4 | 14 | 34 | Ovary | Low grade serous carcinoma | T1bN0M0 | - | IB | Malignant |
| B5 | 15 | 56 | Ovary | High grade serous carcinoma | T2N0M0 | - | II | Malignant |
| B6 | 16 | 22 | Ovary | High grade serous carcinoma | T2bN0M0 | - | IIB | Malignant |
| B7 | 17 | 33 | Ovary | High grade serous carcinoma | T1N0M0 | - | I | Malignant |
| B8 | 18 | 56 | Ovary | High grade serous carcinoma | T1N0M0 | - | I | Malignant |
| B9 | 19 | 48 | Ovary | High grade serous carcinoma | T1N0M0 | - | I | Malignant |
| B10 | 20 | 43 | Ovary | High grade serous carcinoma | T1N0M0 | - | I | Malignant |
| C1 | 21 | 48 | Ovary | High grade serous carcinoma | T2bN0M0 | - | IIB | Malignant |
| C2 | 22 | 51 | Ovary | High grade serous carcinoma | T3cN1M0 | - | IIIC | Malignant |
| C3 | 23 | 42 | Ovary | High grade serous carcinoma | T1N0M0 | - | I | Malignant |
| C4 | 24 | 47 | Ovary | High grade serous carcinoma | T3N0M0 | - | III | Malignant |
| C5 | 25 | 64 | Ovary | High grade serous carcinoma | T1N0M0 | - | I | Malignant |
| C6 | 26 | 52 | Ovary | High grade serous carcinoma | T1aN0M0 | - | IA | Malignant |
| C7 | 27 | 53 | Ovary | High grade serous carcinoma | T2N0M1 | - | IV | Malignant |
| C8 | 28 | 60 | Ovary | High grade serous carcinoma | T1bN0M0 | - | IB | Malignant |
| C9 | 29 | 54 | Ovary | High grade serous carcinoma | T3cN1M0 | - | IIIC | Malignant |
| C10 | 30 | 53 | Ovary | High grade serous carcinoma | T1N0M0 | - | I | Malignant |
| D1 | 31 | 47 | Ovary | High grade serous carcinoma | T1aN0M0 | - | IA | Malignant |
| D2 | 32 | 48 | Ovary | High grade serous carcinoma | T1N0M0 | - | I | Malignant |
| D3 | 33 | 48 | Ovary | High grade serous carcinoma | T3cN0M0 | - | IIIC | Malignant |
| D4 | 34 | 53 | Ovary | High grade serous carcinoma | T1cN0M0 | - | IC | Malignant |
| D5 | 35 | 26 | Ovary | High grade serous carcinoma | T3cN1M0 | - | IIIC | Malignant |
| D6 | 36 | 35 | Ovary | High grade serous carcinoma | T1aN0M0 | - | IA | Malignant |
| D7 | 37 | 58 | Ovary | High grade serous carcinoma | T1N0M0 | - | I | Malignant |
| D8 | 38 | 60 | Ovary | High grade serous carcinoma | T1aN0M0 | - | IA | Malignant |
| D9 | 39 | 55 | Ovary | High grade serous carcinoma | T1N0M0 | - | I | Malignant |
| D10 | 40 | 67 | Ovary | High grade serous carcinoma | T1aN0M0 | - | IA | Malignant |
| E1 | 41 | 57 | Ovary | High grade serous carcinoma | T3cN1M0 | - | IIIC | Malignant |
| E2 | 42 | 41 | Ovary | High grade serous carcinoma | T1N0M0 | - | I | Malignant |
| E3 | 43 | 63 | Ovary | High grade serous carcinoma with necrosis | T1N0M0 | - | I | Malignant |
| E4 | 44 | 52 | Ovary | High grade serous carcinoma | T2N0M0 | - | II | Malignant |
| E5 | 45 | 66 | Ovary | High grade serous carcinoma | T3N1M0 | - | IIIC | Malignant |
| E6 | 46 | 52 | Ovary | High grade serous carcinoma | T1cN0M0 | - | IC | Malignant |
| E7 | 47 | 64 | Ovary | High grade serous carcinoma | T3N1M0 | - | IIIC | Malignant |
| E8 | 48 | 62 | Ovary | High grade serous carcinoma | T2N0M0 | - | II | Malignant |
| E9 | 49 | 42 | Ovary | High grade serous carcinoma | T2N0M0 | - | II | Malignant |
| E10 | 50 | 49 | Ovary | High grade serous carcinoma | T2N0M0 | - | II | Malignant |
| F1 | 51 | 59 | Ovary | High grade serous carcinoma with necrosis | T1cN0M0 | - | IC | Malignant |
| F2 | 52 | 42 | Ovary | High grade serous carcinoma (sparse) | T3cN1M0 | - | IIIC | Malignant |
| F3 | 53 | 49 | Ovary | High grade serous carcinoma | T1aN0M0 | - | IA | Malignant |
| F4 | 54 | 69 | Ovary | High grade serous carcinoma | T2N0M0 | - | II | Malignant |
| F5 | 55 | 42 | Ovary | High grade serous carcinoma | T3N1M0 | - | IIIC | Malignant |
| F6 | 56 | 53 | Ovary | High grade serous carcinoma with necrosis | T1aN0M0 | - | IA | Malignant |
| F7 | 57 | 47 | Ovary | High grade serous carcinoma | T1cN0M0 | - | IC | Malignant |
| F8 | 58 | 49 | Ovary | High grade serous carcinoma | T3N1M0 | - | IIIC | Malignant |
| F9 | 59 | 52 | Ovary | High grade serous carcinoma | T3cN1M0 | - | IIIC | Malignant |
| F10 | 60 | 55 | Ovary | High grade serous carcinoma | T3N1M0 | - | IIIC | Malignant |
| G1 | 61 | 52 | Ovary | High grade serous carcinoma | T2N0M0 | - | II | Malignant |
| G2 | 62 | 51 | Ovary | High grade serous carcinoma | T2N0M0 | - | II | Malignant |
| G3 | 63 | 41 | Ovary | High grade serous carcinoma | T1aN0M0 | - | IA | Malignant |
| G4 | 64 | 56 | Ovary | High grade serous carcinoma | T3N0M0 | - | III | Malignant |
| G5 | 65 | 55 | Ovary | High grade serous carcinoma | T1N0M0 | - | I | Malignant |
| G6 | 66 | 48 | Ovary | High grade serous carcinoma | T3aN0M0 | - | IIIA | Malignant |
| G7 | 67 | 60 | Ovary | High grade serous carcinoma | T2bN0M0 | - | IIB | Malignant |
| G8 | 68 | 51 | Ovary | High grade serous carcinoma | T1aN0M0 | - | IA | Malignant |
| G9 | 69 | 46 | Ovary | Mucinous papillary adenocarcinoma (necrosis) | T1aN0M0 | * | IA | Malignant |
| G10 | 70 | 49 | Ovary | Endometrioid adenocarcinoma | T1N0M0 | 2 | I | Malignant |
| H1 | 71 | 34 | Ovary | Mucinous adenocarcinoma | T1bN0M0 | 2 | IB | Malignant |
| H2 | 72 | 37 | Ovary | Mucinous adenocarcinoma | T1aN0M0 | 2 | IA | Malignant |
| H3 | 73 | 39 | Ovary | Mucinous adenocarcinoma with necrosis | T1aN0M0 | 2 | IA | Malignant |
| H4 | 74 | 54 | Ovary | Mucinous adenocarcinoma | T2aN0M0 | 2 | IIA | Malignant |
| H5 | 75 | 41 | Ovary | Mucinous adenocarcinoma with necrosis | T1bN0M0 | 2--3 | IB | Malignant |
| H6 | 76 | 50 | Ovary | Mucinous adenocarcinoma | T3cN1M0 | 3 | IIIC | Malignant |
| H7 | 77 | 52 | Ovary | Mucinous adenocarcinoma | T1bN0M0 | 3 | IB | Malignant |
| H8 | 78 | 38 | Ovary | Mucinous adenocarcinoma | T1N0M0 | 3 | I | Malignant |
| H9 | 79 | 29 | Ovary | Mucinous adenocarcinoma | T3N0M0 | 3 | III | Malignant |
| H10 | 80 | 58 | Ovary | Endometrioid adenocarcinoma | T1aN0M0 | 3 | IA | Malignant |
| I1 | 81 | 47 | Lymph node | Metastatic serous carcinoma from ovary | - | - | - | Metastasis |
| I2 | 82 | 48 | Lymph node | Metastatic serous carcinoma from ovary | - | - | - | Metastasis |
| I3 | 83 | 46 | Lymph node | Metastatic serous carcinoma from ovary | | - | - | Metastasis |
| I4 | 84 | 54 | Lymph node | Metastatic serous carcinoma from ovary | - | - | - | Metastasis |
| I5 | 85 | 83 | Lymph node | Metastatic serous carcinoma from ovary | - | * | - | Metastasis |
| I6 | 86 | 48 | Lymph node | Metastatic clear cell carcinoma from ovary | - | - | - | Metastasis |
| I7 | 87 | 56 | Pelvic cavity | Metastatic serous carcinoma of fibrofatty tissue from ovary of No.64 | - | - | - | Metastasis |
| I8 | 88 | 57 | Greater omentum | Metastatic serous carcinoma from ovary | - | - | - | Metastasis |
| I9 | 89 | 50 | Lymph node | Metastatic serous carcinoma from ovary | - | - | - | Metastasis |
| I10 | 90 | 53 | Pelvic cavity | Metastatic serous carcinoma of fibrofatty tissue from ovary | - | - | - | Metastasis |
| J1 | 91 | 69 | Ovary | Adjacent normal ovary tissue | - | - | - | NAT |
| J2 | 92 | 48 | Ovary | Adjacent normal ovary tissue | - | - | - | NAT |
| J3 | 93 | 53 | Ovary | Adjacent normal ovary tissue | - | - | - | NAT |
| J4 | 94 | 42 | Ovary | Adjacent normal ovary tissue | - | - | - | NAT |
| J5 | 95 | 42 | Ovary | Adjacent normal ovary tissue | - | - | - | NAT |
| J6 | 96 | 40 | Ovary | Adjacent normal ovary tissue | - | - | - | NAT |
| J7 | 97 | 59 | Ovary | Adjacent normal ovary tissue | - | - | - | NAT |
| J8 | 98 | 42 | Ovary | Adjacent normal ovary tissue | - | - | - | NAT |
| J9 | 99 | 35 | Ovary | Adjacent normal ovary tissue | - | - | - | NAT |
| J10 | 100 | 45 | Ovary | Adjacent normal ovary tissue | - | - | - | NAT |
| – | – | M | 42 | Adrenal gland | Pheochromocytoma (tissue marker) | – |  |  |


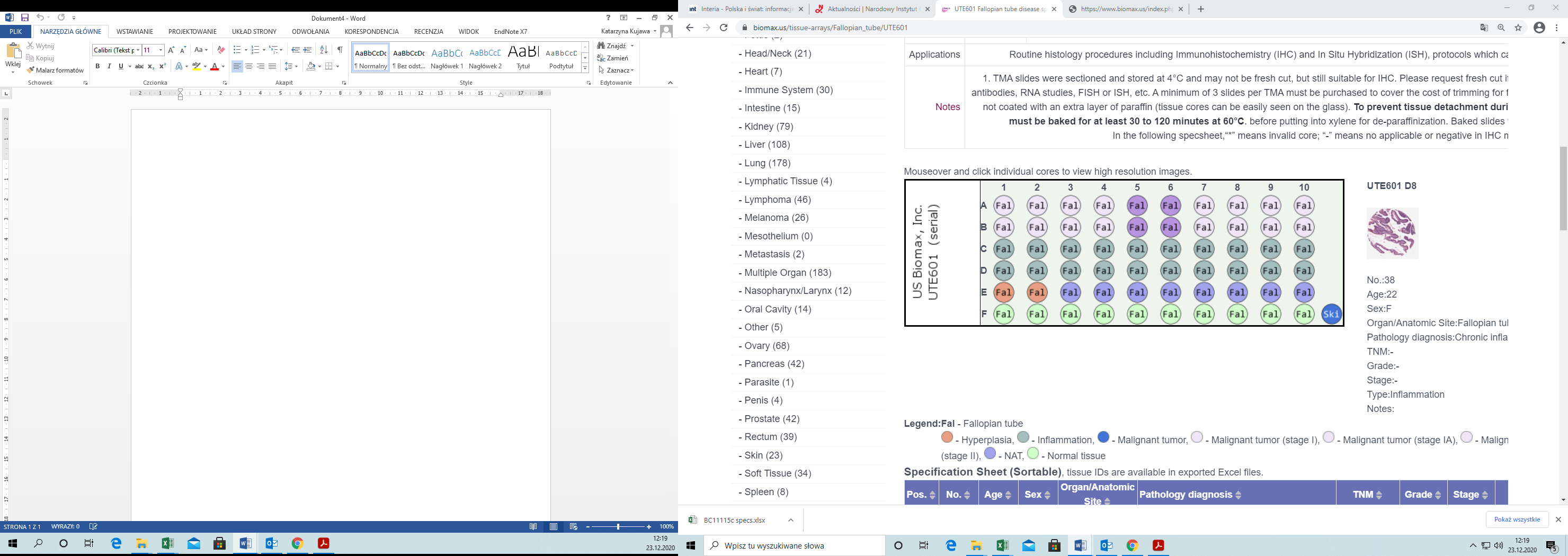
UTE601


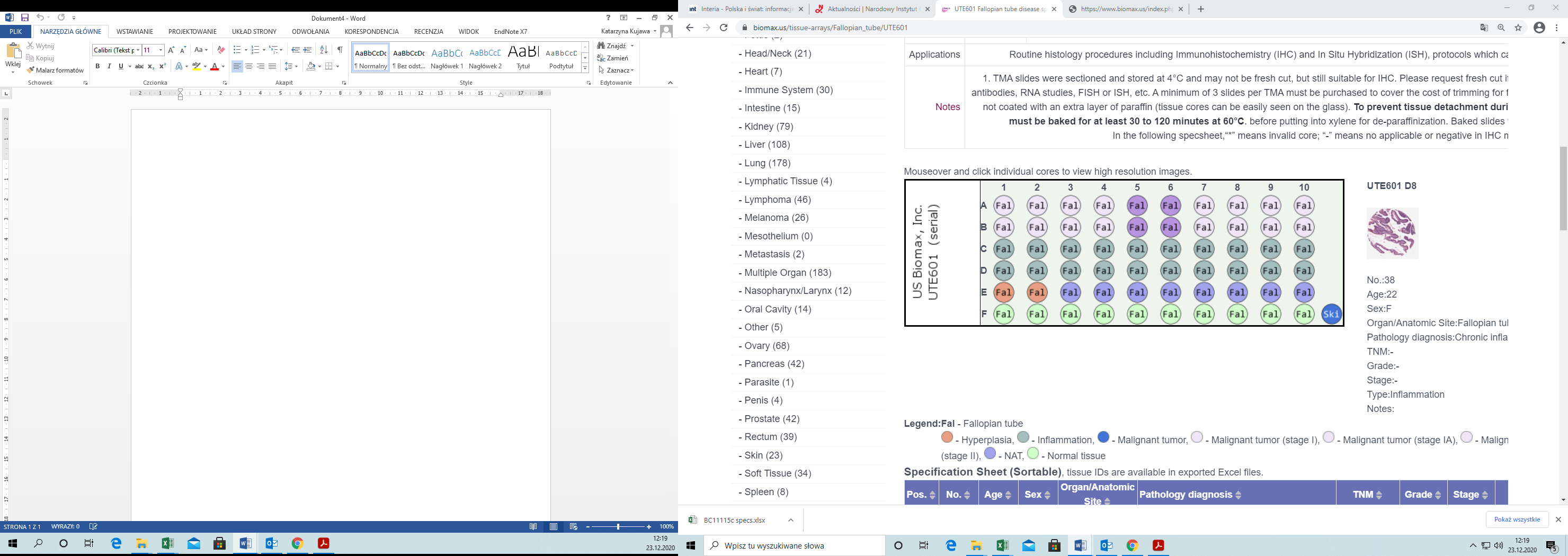


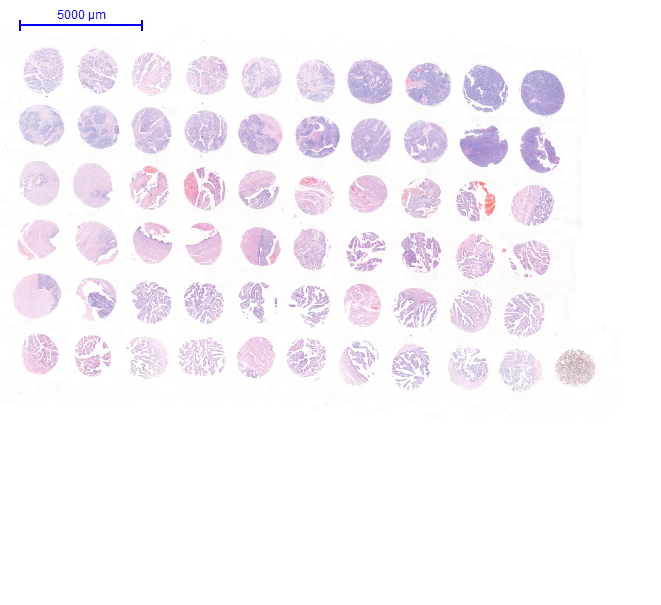


**H+E UTE601 tissue array**

**Figure S7.** “Fallopian tube disease spectrum” UTE601, US Biomax.

**Table S7.** US Biomax, UTE601.

|  | | | | | | | |
| --- | --- | --- | --- | --- | --- | --- | --- |
| [**Pos**](http://www.biomax.us/) | [**No.**](http://www.biomax.us/) | [**Sex**](http://www.biomax.us/) | [**Age**](http://www.biomax.us/) | [**Organ**](http://www.biomax.us/) | [**Pathology diagnosis**](http://www.biomax.us/) | [**Grade**](http://www.biomax.us/) | [**Type †**](http://www.biomax.us/) |
| A1 | 1 | F | 47 | Fallopian tube | Adenocarcinoma | 2 | Malignant |
| A2 | 2 | F | 47 | Fallopian tube | Adenocarcinoma | 2 | Malignant |
| A3 | 3 | F | 46 | Fallopian tube | Adenocarcinoma | 3 | Malignant |
| A4 | 4 | F | 46 | Fallopian tube | Adenocarcinoma | 3 | Malignant |
| A5 | 5 | F | 43 | Fallopian tube | Adenocarcinoma | 2–3 | Malignant |
| A6 | 6 | F | 43 | Fallopian tube | Adenocarcinoma | 2–3 | Malignant |
| A7 | 7 | F | 52 | Fallopian tube | Adenocarcinoma | 3 | Malignant |
| A8 | 8 | F | 52 | Fallopian tube | Adenocarcinoma | 3 | Malignant |
| A9 | 9 | F | 53 | Fallopian tube | Adenocarcinoma | 3 | Malignant |
| A10 | 10 | F | 53 | Fallopian tube | Adenocarcinoma | 3 | Malignant |
| B1 | 11 | F | 60 | Fallopian tube | Adenocarcinoma | 3 | Malignant |
| B2 | 12 | F | 60 | Fallopian tube | Adenocarcinoma | 3 | Malignant |
| B3 | 13 | F | 40 | Fallopian tube | Adenocarcinoma | 3 | Malignant |
| B4 | 14 | F | 40 | Fallopian tube | Adenocarcinoma | 3 | Malignant |
| B5 | 15 | F | 57 | Fallopian tube | Adenocarcinoma | 3 | Malignant |
| B6 | 16 | F | 57 | Fallopian tube | Adenocarcinoma | 3 | Malignant |
| B7 | 17 | F | 51 | Fallopian tube | Adenocarcinoma | 3 | Malignant |
| B8 | 18 | F | 51 | Fallopian tube | Adenocarcinoma | 3 | Malignant |
| B9 | 19 | F | 53 | Fallopian tube | Adenocarcinoma | 3 | Malignant |
| B10 | 20 | F | 53 | Fallopian tube | Adenocarcinoma | 3 | Malignant |
| C1 | 21 | F | 41 | Fallopian tube | Chronic inflammation (of smooth muscle) | – | Inflammation |
| C2 | 22 | F | 41 | Fallopian tube | Chronic inflammation (of smooth muscle) | – | Inflammation |
| C3 | 23 | F | 32 | Fallopian tube | Chronic inflammation | – | Inflammation |
| C4 | 24 | F | 32 | Fallopian tube | Chronic inflammation | – | Inflammation |
| C5 | 25 | F | 46 | Fallopian tube | Chronic inflammation | – | Inflammation |
| C6 | 26 | F | 46 | Fallopian tube | Chronic inflammation (of fibrous tissue and smooth muscle) | – | Inflammation |
| C7 | 27 | F | 34 | Fallopian tube | Chronic inflammation (of fibrous tissue and smooth muscle) | – | Inflammation |
| C8 | 28 | F | 34 | Fallopian tube | Chronic inflammation | – | Inflammation |
| C9 | 29 | F | 34 | Fallopian tube | Chronic inflammation | – | Inflammation |
| C10 | 30 | F | 34 | Fallopian tube | Chronic inflammation | – | Inflammation |
| D1 | 31 | F | 53 | Fallopian tube | Chronic inflammation (of fibrous tissue and smooth muscle) | – | Inflammation |
| D2 | 32 | F | 53 | Fallopian tube | Chronic inflammation | – | Inflammation |
| D3 | 33 | F | 49 | Fallopian tube | Chronic inflammation | – | Inflammation |
| D4 | 34 | F | 49 | Fallopian tube | Chronic inflammation | – | Inflammation |
| D5 | 35 | F | 49 | Fallopian tube | Chronic inflammation | – | Inflammation |
| D6 | 36 | F | 49 | Fallopian tube | Chronic inflammation | – | Inflammation |
| D7 | 37 | F | 22 | Fallopian tube | Chronic inflammation | – | Inflammation |
| D8 | 38 | F | 22 | Fallopian tube | Chronic inflammation | – | Inflammation |
| D9 | 39 | F | 29 | Fallopian tube | Chronic inflammation | – | Inflammation |
| D10 | 40 | F | 29 | Fallopian tube | Chronic inflammation | – | Inflammation |
| E1 | 41 | F | 27 | Fallopian tube | Chronic inflammation with hyperplasia of epithelium | – | Hyperplasia |
| E2 | 42 | F | 27 | Fallopian tube | Chronic inflammation with hyperplasia of epithelium | – | Hyperplasia |
| E3 | 43 | F | 56 | Fallopian tube | Cancer adjacent normal oviductal tissue | – | NAT |
| E4 | 44 | F | 56 | Fallopian tube | Cancer adjacent normal oviductal tissue | – | NAT |
| E5 | 45 | F | 34 | Fallopian tube | Cancer adjacent normal oviductal tissue | – | NAT |
| E6 | 46 | F | 34 | Fallopian tube | Cancer adjacent normal oviductal tissue | – | NAT |
| E7 | 47 | F | 26 | Fallopian tube | Cancer adjacent normal oviductal tissue (fibrous tissue and smooth muscle) | – | NAT |
| E8 | 48 | F | 26 | Fallopian tube | Cancer adjacent normal oviductal tissue | – | NAT |
| E9 | 49 | F | 47 | Fallopian tube | Cancer adjacent normal oviductal tissue | – | NAT |
| E10 | 50 | F | 47 | Fallopian tube | Cancer adjacent normal oviductal tissue | – | NAT |
| F1 | 51 | F | 21 | Fallopian tube | Normal oviductal tissue | – | Normal |
| F2 | 52 | F | 21 | Fallopian tube | Normal oviductal tissue | – | Normal |
| F3 | 53 | F | 15 | Fallopian tube | Normal oviductal tissue | – | Normal |
| F4 | 54 | F | 15 | Fallopian tube | Normal oviductal tissue | – | Normal |
| F5 | 55 | F | 18 | Fallopian tube | Normal oviductal tissue | – | Normal |
| F6 | 56 | F | 18 | Fallopian tube | Normal oviductal tissue | – | Normal |
| F7 | 57 | F | 21 | Fallopian tube | Normal oviductal tissue | – | Normal |
| F8 | 58 | F | 21 | Fallopian tube | Normal oviductal tissue | – | Normal |
| F9 | 59 | F | 21 | Fallopian tube | Normal oviductal tissue | – | Normal |
| F10 | 60 | F | 21 | Fallopian tube | Normal oviductal tissue | – | Normal |
| – | – | M | 58 | Skin | Malignant melanoma (tissue marker) | – | Malignant |


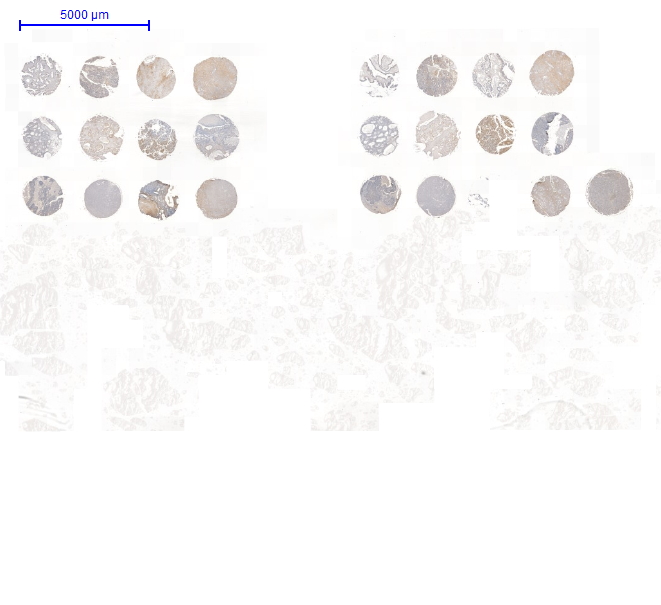


MFAP5 immunohistochemistry in T112b tissue array (test array)

**Figure S8.** Immunohistochemical detection of MFAP5 in T112b tissue array. Pannoramic 250 Flash II Scanner, scale bar: 5000 µm.


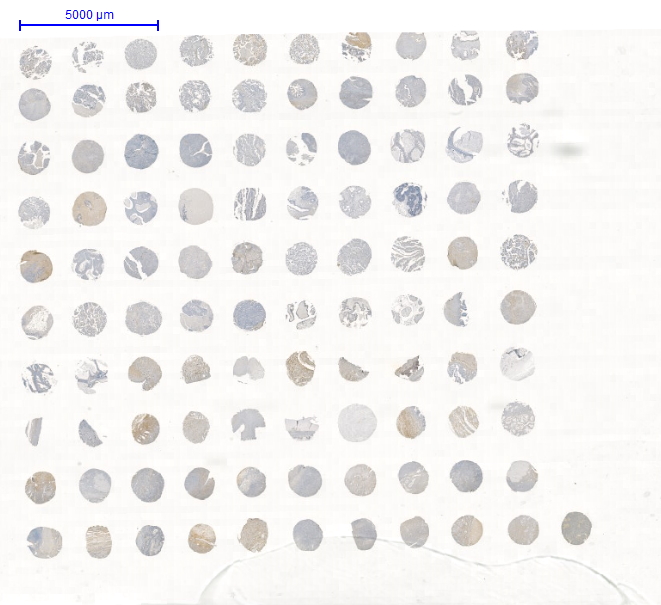


MFAP5 immunohistochemistry in OV1005a tissue array

Malignant

Metastasis

Borderline

Benign

Cancer adjacent normal ovary tissue

Pheochrom

ocytoma

Metastasis

Borderline

Normal tissue

**Figure S9.** Immunohistochemical detection of MFAP5 in OV1005a tissue array. Pannoramic 250 Flash II Scanner, scale bar: 5000 µm.


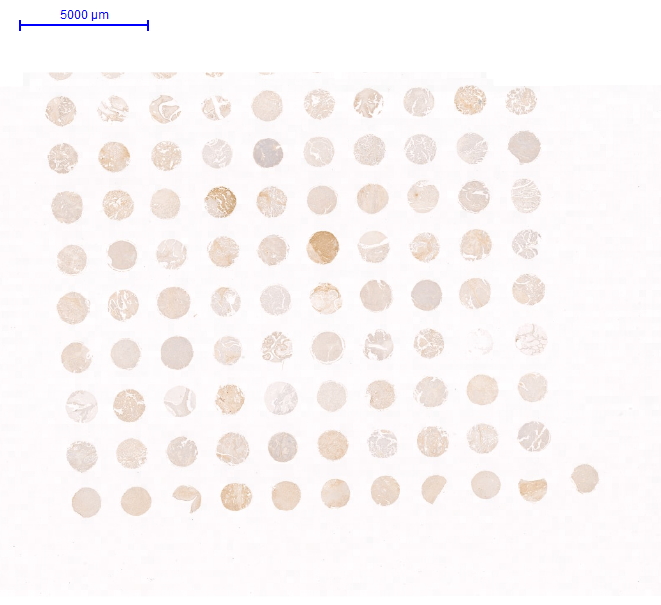


mucinous

Metastasis

endometrioid

mucinous

Cancer adjacent normal ovary tissue

Pheochro

mocytoma

high grade serous carcinoma

low grade serous carcinoma

endometrioid

MFAP5 immunohistochemistry in BC11115c tissue array

**Figure S10.** Immunohistochemical detection of MFAP5 in BC11115c tissue array. Pannoramic 250 Flash II Scanner, scale bar: 5000 µm.


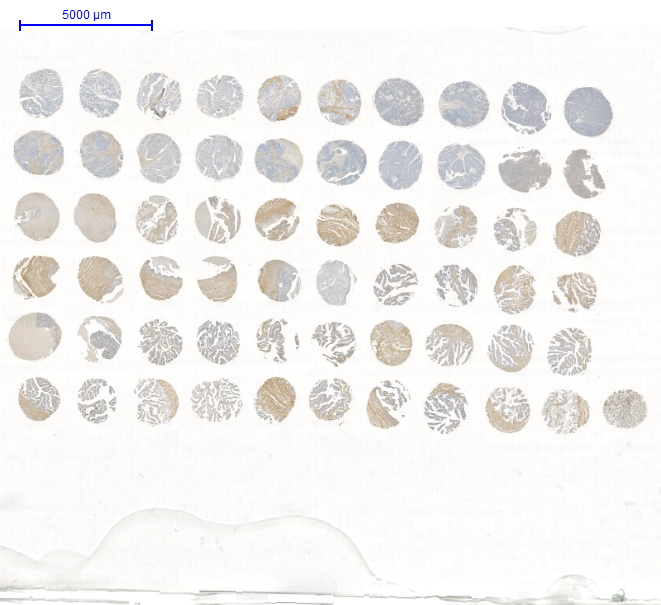


MFAP5 immunohistochemistry in UTE601 tissue array

Malignant

Inflammation

Adjacent normal oviductal tissue

Malignant melanoma

Normal tissue

**Figure S11.** Immunohistochemical detection of MFAP5 in UTE601 tissue array. Pannoramic 250 Flash II Scanner, scale bar: 5000 µm.

**Evaluation of antibodies**

Western blot evaluation of anti-MFAP5 Ab.


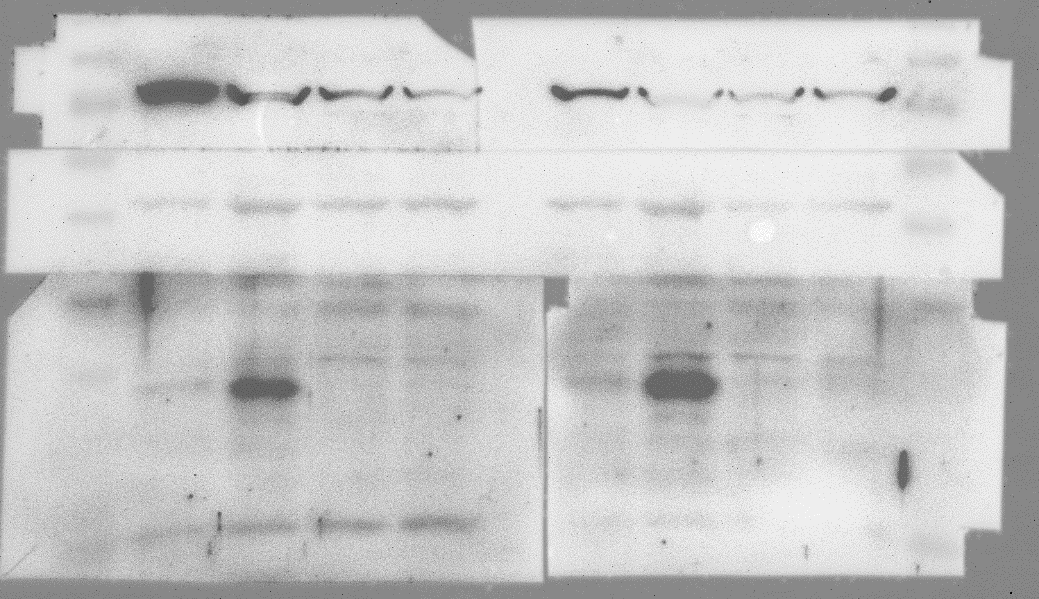


**kDa**

**55**

**40**

**35**

**25**

**15**

U2OS

ES2

MFAP5

ES2

pLNCX2

ES2

beta actin (42 kDa)

**MFAP5 (~20 kDa)**

**Figure S12.** Results of Western blot using 15727-1-AP antibody (1:1000 dilution) for MFAP5 detection. U2OS was used as a positive control (cell line with natural expression of MFAP5). ES2 cell line was modified by retroviral transfer system to overexpress MFAP5 (marked “ES2 MFAP5”). “ES2 pLNCX2” is ES2 a control cell line modified using empty vector (pLNCX2). ES2 denotes wild type cell line. We could notice strong signal for MFAP5 expression in modified ES2 cell line. U2OS exhibits signal for MFAP5 expression, but weaker than ES2-MFAP5.

We performed WB evaluation of 15727-1-AP antibody because producer did not show WB results for this antibody.

Immunohistochemical evaluation of anti-MFAP5 antibody

Positive and negative control tissues for MFAP5 staining were chosen based on the Human Protein Atlas database. Control tissues were selected from those known to be either positive (placenta, colon) or negative (ovary) for MFAP5 expression within specified morphologic structures. This allowed us to verify specificity of the analyzed antibody.

**Figure S13.** Immunohistochemical staining of the ovary tissues with anti-MFAP5 antibody 15727-1-AP (Proteintech), dilution 1:400. Left image presents cross-section of the ovary and right image presents stroma of the ovary which is negative in regard to MFAP5. Pannoramic 250 Flash II Scanner, scale bar 10000 µm (left image) and 500 µm (right image).


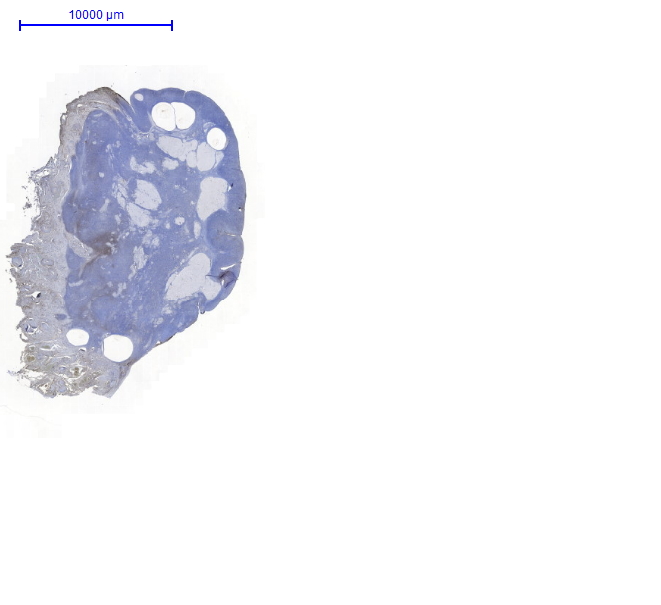

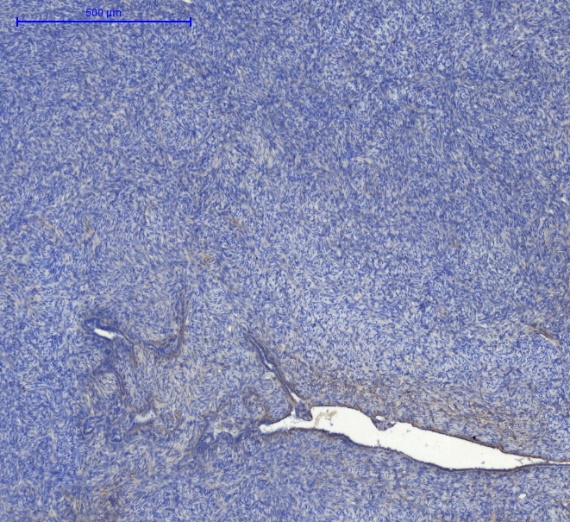


**Figure S14.** Immunohistochemical staining of control tissues with anti-MFAP5 antibody 15727-1-AP (Proteintech), dilution 1:400. **A.** Staining of placenta tissue: visible stained elements of syncytiotrophoblast. **B.** Staining of colon tissue: visible unstained glandular cells (marked by arrows) and stained connective tissue fibers around. Pannoramic 250 Flash II Scanner, scale bar 200 µm (upper image) and 100 µm (lower image).


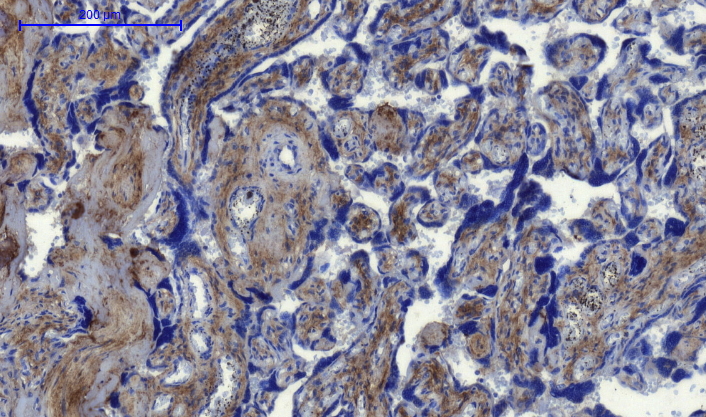


**A.** Anti-MFAP5 staining in placenta tissue

**B.** Anti-MFAP5 staining in colon tissue


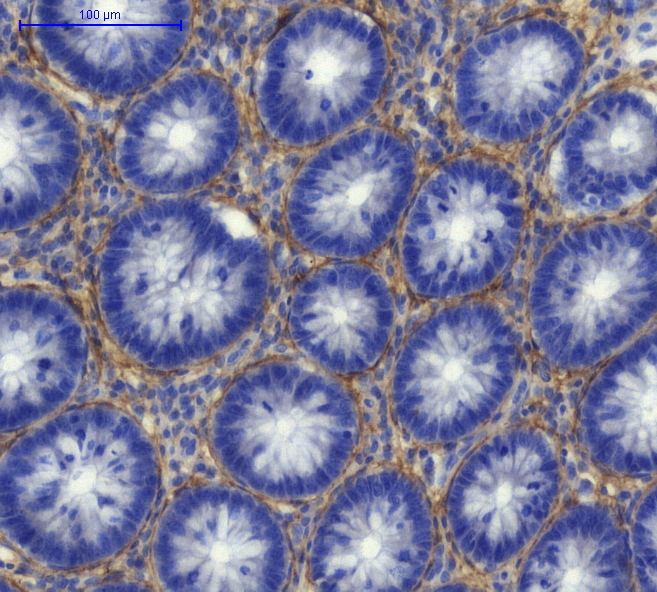

Supplement: Supplementary file 1 [file ijms-23-15994-s001.zip › ijms-2000186-supplementary.docx]
